# Design criteria for stable Pt/C fuel cell catalysts

**DOI:** 10.3762/bjnano.5.5

**Published:** 2014-01-16

**Authors:** Josef C Meier, Carolina Galeano, Ioannis Katsounaros, Jonathon Witte, Hans J Bongard, Angel A Topalov, Claudio Baldizzone, Stefano Mezzavilla, Ferdi Schüth, Karl J J Mayrhofer

**Affiliations:** 1 Department of Interface Chemistry and Surface Engineering, Max-Planck-Institut für Eisenforschung GmbH, Max-Planck-Strasse 1, 40237 Düsseldorf, Germany; 2 Department of Heterogeneous Catalysis, Max-Planck-Institut für Kohlenforschung, Kaiser-Wilhelm-Platz 1, 45470 Mülheim an der Ruhr, Germany

**Keywords:** catalyst design criteria, degradation mechanisms, fuel cell catalyst, nanoparticles, stability

## Abstract

Platinum and Pt alloy nanoparticles supported on carbon are the state of the art electrocatalysts in proton exchange membrane fuel cells. To develop a better understanding on how material design can influence the degradation processes on the nanoscale, three specific Pt/C catalysts with different structural characteristics were investigated in depth: a conventional Pt/Vulcan catalyst with a particle size of 3–4 nm and two Pt@HGS catalysts with different particle size, 1–2 nm and 3–4 nm. Specifically, Pt@HGS corresponds to platinum nanoparticles incorporated and confined within the pore structure of the nanostructured carbon support, i.e., hollow graphitic spheres (HGS). All three materials are characterized by the same platinum loading, so that the differences in their performance can be correlated to the structural characteristics of each material. The comparison of the activity and stability behavior of the three catalysts, as obtained from thin film rotating disk electrode measurements and identical location electron microscopy, is also extended to commercial materials and used as a basis for a discussion of general fuel cell catalyst design principles. Namely, the effects of particle size, inter-particle distance, certain support characteristics and thermal treatment on the catalyst performance and in particular the catalyst stability are evaluated. Based on our results, a set of design criteria for more stable and active Pt/C and Pt-alloy/C materials is suggested.

## Introduction

The hydrogen-fueled proton exchange membrane fuel cell (PEMFC) is a promising technology for energy conversion especially for local or portable applications [1]. PEMFC convert chemical energy stored in hydrogen into electrical energy in an electrochemical process that requires efficient catalysts for both the facile hydrogen oxidation reaction (HOR) at the anode side as well as the more sluggish oxygen reduction reaction (ORR) at the cathode side of the fuel cell [2]. The state of the art electrocatalyst for both electrodes are Pt or Pt-alloys dispersed in the form of nanoparticles on a carbon support, in order to achieve a maximum of active sites. Practical performance, however, not only demands high activities per mass for the ORR, but also stability against the aggressive conditions that occur in the fuel cell under operation, particularly on the cathode side [3]. While significant knowledge on factors that influence the activity of the catalyst was obtained in recent years such as alloying platinum with transition metals [4–7] or varying the particle size [8–14], many questions regarding the fundamental degradation mechanisms of such systems remain [15–16].

**Figure 1:**
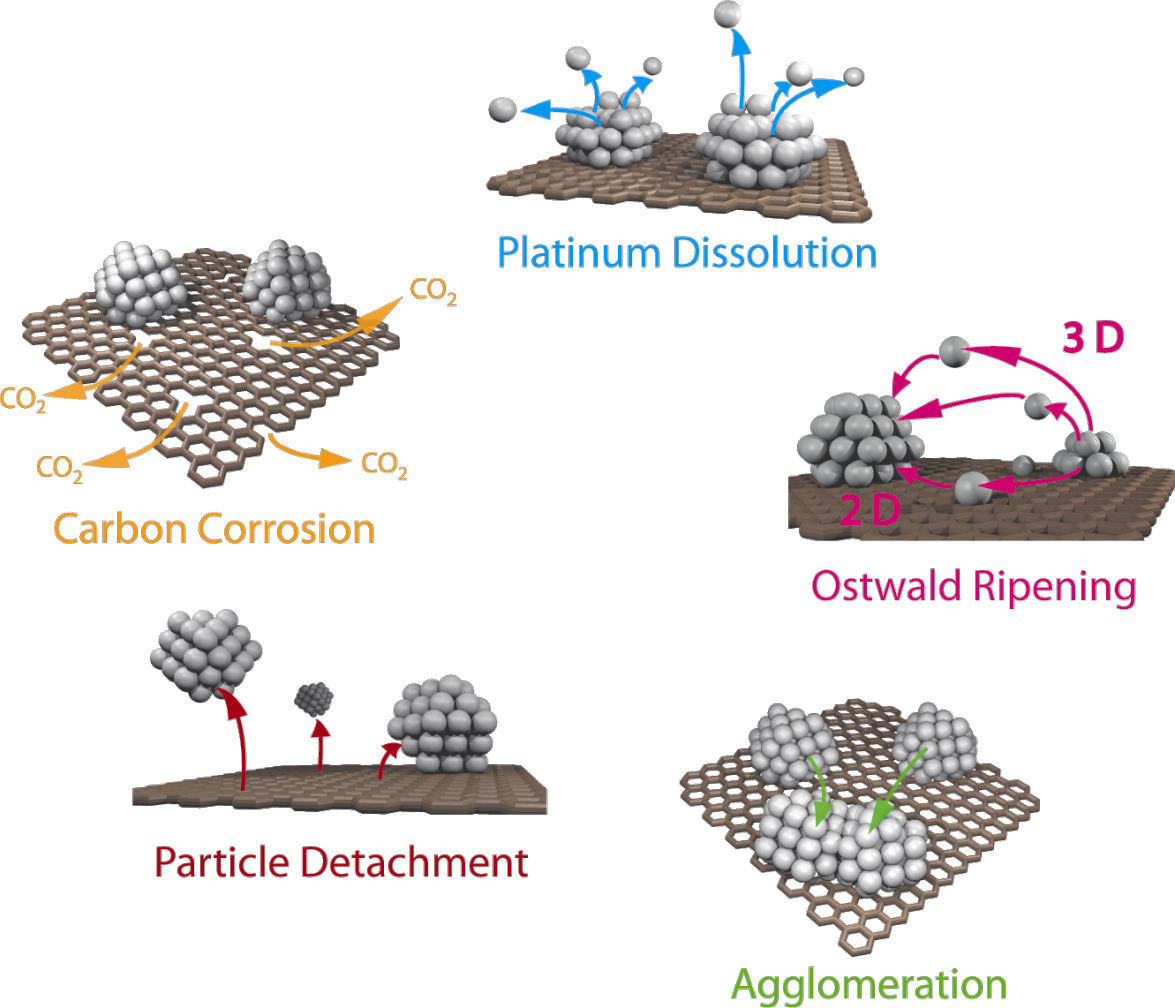
Simplified representation of suggested degradation mechanisms for platinum particles on a carbon support in fuel cells.

A stable fuel cell catalyst needs to preserve its activity over an extended lifetime and to avoid degradation under operation, which is macroscopically reflected in a loss of the electrochemically active surface area (ECSA). A gradual loss of ECSA will inevitably lead to efficiency losses of the fuel cell and can eventually reach an unacceptable level, thus determining the end of fuel cell life as a whole. The degradation of fuel cell catalysts depends on multiple parameters linked to the operation conditions of the cell as well as the structure and composition of the electrocatalyst material. Temperature, pH value, potential as well as the humidity and purity of fuel and oxidant feeds are just a few operation parameters that influence the degradation behavior of the catalyst [3,17]. One mode of operation that was found to be particularly harmful for the electrocatalyst are start-up/shut-down conditions, as they can lead to severe potential changes at the cathode, which result in a rapid degradation of the catalyst [17–22]. Over recent years, attempts to circumvent the severe loss of ECSA and the degradation under various conditions have been mainly based on approaches to enhance materials, predominantly by modifying the properties of the support. Finding substitutes for the commonly used carbon black supports is a demanding task, as only few materials present similar electronic conductivities in combination with high surface areas and comparable chemical inertness at the same time [23]. Due to the large versatility of carbon structures, many research groups have focused on a variety of alternative carbon materials [24] as supports for fuel cell applications such as single walled and multi-walled carbon nanotubes (SWCNTs, MWCNTs) [25–26], graphene [27], carbon nanofibers [28], nanohorns [29], ordered mesoporous carbons (OMCs) [30–31], carbon aerogels [32], carbon shells [33–36], colloid-imprinted carbon supports (CIC) [37] and even boron-doped diamond structures [38–39]. Alternatively, certain non-carbon materials (e.g., oxides, carbides and nitrides of metals such as Ti, W, Mo etc.) exhibit promising corrosion resistances under fuel cell conditions. However, most of these non-carbon materials suffer from low conductivities and/or poor platinum dispersion, thus limiting the efficiency of the fuel cell [17]. Despite the demonstration of an improved stability as electrocatalyst under particular conditions, a general and comprehensive performance gain for these advanced materials is still missing. Moreover, in most cases no detailed understanding is available on how the material design influences the degradation pathways that are responsible for the macroscopically observed platinum surface area loss. For standard Pt/C catalysts, indications for a variety of different degradation mechanisms are reported and summarized in the literature [3,15–17,40–42]. In Figure 1 we provide a short summary of catalyst degradation mechanisms that have been suggested to occur in hydrogen fuel cells.

A variety of studies have demonstrated that **platinum dissolution** can occur in PEMFCs during operation, as dissolved platinum was detected in the water stream that exited the fuel cells [43]. Platinum was also found to redeposit in the membrane of PEMFCs as a consequence of the reduction with hydrogen that permeates the ionomer from the anode chamber of the cell [44–46]. Platinum dissolution is expected to be especially severe for smaller platinum particles, which have a higher surface energy and are thus considered to dissolve already at lower potentials than bulk platinum (Gibbs–Thomson effect) [14–15]. If the dissolved platinum is redeposited on larger platinum particles, significant particle growth can occur and the according degradation mechanism is called **Ostwald ripening** (3D Ostwald ripening, if the dissolved platinum species travel through the electrolyte, and 2D Ostwald ripening – as known from high temperature TEM studies in the absence of an electrolyte – if platinum atoms are believed to diffuse along the carbon support) [44–45,47–48]. Another possible explanation for the growth of platinum particles in the catalyst layer is **coalescence** [17,49]. This may be either due to migration and collision of platinum particles on the surface of the carbon support with successive coalescence, or due to strong carbon corrosion. In the second case, neighboring but initially separated particles come into contact with each other because of a successive shrinkage of the carbon support on which they are located [49]. However, also in the first possible case of agglomeration and coalescence due to migration, carbon corrosion may be involved and lead to a weakening of the interactions between platinum particles and support. Alternatively a preferential local corrosion of the support in the surrounding of the platinum particles may facilitate particle movement [50]. A weakening of the interaction between particle and support due to carbon corrosion is also believed to be the cause for the observed **detachment** of whole platinum particles from the support [51–52]. In this context, the ability of platinum to catalyze the oxidation of carbon was suggested to play a decisive role [50,53]. Finally, severe **carbon corrosion** can lead to a loss of the structural integrity of the catalyst layer, which reduces the porosity and thus can result, besides triggering the above catalyst degradation pathways, in mass transport limitations for the reactants [54]. It is also assumed that the formation of excessive oxygenated functional groups at the carbon surface can increase the hydrophilicity of the support and thus enhance flooding effects that can hamper the transport of oxygen to platinum within the catalyst layer and thus lead to a decline in performance [17]. In the light of the aforementioned reasons for the deterioration of the catalyst performance, it is worth distinguishing conceptually between **primary** and **secondary degradation phenomena**. In this sense, carbon corrosion is a primary degradation process and can be the cause for secondary processes such as platinum particle detachment or agglomeration. Moreover, dissolution of platinum can be considered as another primary degradation phenomenon, which is the precondition for secondary degradation phenomena such as Ostwald ripening or platinum deposition in the ionomer.

Much of the up-to-date knowledge on degradation mechanisms of standard fuel cell catalysts has been derived from post-mortem analyses of membrane electrode assemblies (MEAs) after fuel cell operation. Electron-microscopic techniques played a crucial role in such investigations [44–45,55–58]. However, any catalyst testing within a real fuel cell is time demanding and ambiguous because of the high complexity of cell manufacturing [17,59]. Furthermore, standard electron-microscopic post-mortem analysis demands for extensive statistical evaluations in order to obtain meaningful information. Because of the above reasons, several groups recently highlighted the importance of studying changes in the electrocatalyst microstructure by visualizing one specific catalyst location and its transformation under certain imposed aging conditions. Such investigations follow the example of the well-established high temperature in-situ transmission electron microscopy (TEM) measurements, which is utilized to study the behavior of various kinds of catalyst materials at elevated temperatures [60]. More recently, TEM stability studies under environmental conditions, for instance in moisturized air, were also reported [50],[61–63]. Aiming for a visualization of the degradation processes that electrocatalysts undergo under electrochemical conditions, our group has developed an electron microscopic method to study identical locations of catalysts before and after electrochemical aging (i.e., post mortem) while simulating the operation conditions in a fuel cell [51–52]. The identical location transmission electron microscopy (IL-TEM) approach was recently extended to other electron microscopic techniques, such as scanning electron microscopy (IL-SEM) or electron tomography (IL-tomography) [16,40,64–65], and it has been applied in several studies on the degradation behavior of standard fuel cell catalysts that used accelerated-aging protocols [41–42,49,65–70]. These studies have provided direct visual evidence for all of the above mentioned processes under aggressive potential cycling conditions, namely platinum dissolution [16,68], coalescence [16,41–42], particle detachment [42,51–52], carbon corrosion [16,49,70] and Ostwald ripening (3D) [64]. In many cases, several of the discussed mechanisms were observed to occur simultaneously and their contribution to the overall surface area loss seems to vary depending on the applied protocols and the structural properties of the catalyst [16,42,66–67].

In this study, we use identical location electron microscopic techniques in combination with standard electrochemical techniques to provide an in-depth understanding of degradation phenomena under accelerated-aging conditions at low-temperatures. We investigate the electrochemical activity and the macroscopic as well as nanoscale stability of three different Pt/C materials, i.e., Pt/Vulcan 3–4 nm, Pt@HGS 1–2 nm and Pt@HGS 3–4 nm (with Pt@HGS meaning platinum nanoparticles confined within the carbon support HGS) and compare the results with the performance of other conventional fuel cell catalysts. In this context, we will evaluate possible causes for the previously reported excellent stability of the Pt@HGS 3–4 nm material [71]. We therefore summarize and deepen some of our earlier findings and use them as a basis for a broader discussion about design principles for stable Pt/C materials. In particular the impact of the size of the platinum particles, the inter-particle distances, the structure of the support, and of a thermal treatment will be discussed and we provide guidelines for the design of stable nanostructured fuel cell catalysts.

## Materials

Two HGS-based catalysts, Pt@HGS 1–2 nm and Pt@HGS 3–4 nm, were selected as model nanostructured materials and compared to Pt/Vulcan 3–4 nm. Each of the three materials is characterized by a platinum content of 20 wt %. The comparison of any set of catalysts reveals a different particle size or a different carbon support. While Pt@HGS 1–2 nm and Pt@HGS 3–4 nm both have the same carbon support they can be distinguished by their particle size distribution. Pt@HGS 3–4 nm and Pt/Vulcan 3–4 nm on the other hand offer a comparable particle size distribution, but the structure of the carbon support is different. An in-depth characterization of the degradation behavior of all three materials, thus, promises insight into the effect of both particle size and support structure on the underlying degradation mechanisms.

The hollow graphitic spheres (HGS) provide a mesoporous, three-dimensional interconnected support structure with a high degree of graphitization and a high BET surface area (ca. 1200 m^2^·g^−1^) at the same time. The synthesis was described previously in detail, however a short summary of the synthesis approach is provided in Scheme S1 (Supporting Information File 1). The majority of the pores in the shell of the hollow spheres are in the size range of 3–4 nm while some are larger, about 8–10 nm. The spheres have an average diameter of about 360 nm with an average shell thickness of approximately 50 nm. The mesoporous network is intended to provide a good separation and encapsulation of the particles without losing accessibility to the Pt particles. The access is also facilitated by the short diffusion pathways through the shell, which are a result of the large void in the middle of the sphere. Graphitization is intended to slow down carbon corrosion and the high specific carbon surface area in combination with the three dimensional network is intended to offer large inter-particle distances and a good particle separation [71].

Because of the large surface area and the mesoporous structure, the platinum deposition results in a high dispersion of platinum particles within the network with platinum nanoparticles in a size range of approximately 1–2 nm (Pt@HGS 1–2 nm). An increase in particle size was possible through a thermal-treatment step up to 900 °C for several hours. The annealing step results in a minor sintering of the initial clusters and leads to an average particle size of about 3–4 nm, which is in the range of the pore size distribution. The particle growth is remarkably well-controlled in the mesoporous network and gives a monodisperse distribution of the particle sizes after the sintering (Pt@HGS 3–4 nm). A first investigation of the electrochemical properties of the Pt@HGS 3–4 nm material has been described before [71]. The synthesis of the Pt/Vulcan 3–4 nm material was also described previously [16]. Contrary to the HGS-based catalysts, colloidal deposition was utilized for platinum deposition in the Pt/Vulcan material. This allowed us to obtain a comparable and defined particle size distribution to the one of Pt@HGS 3–4 nm. Another Pt/Vulcan material with an average particle size of 5–6 nm was used for the activity comparison and was manufactured by mild thermal treatment of the Pt/Vulcan 3–4 nm material.

## Results and Discussion

### Activity of Pt/C materials

Before focusing on stability, it is essential to compare the activity of the synthesized materials for the ORR (Pt@HGS 1–2 nm, Pt@HGS 3–4 nm and Pt/Vulcan 3–4 nm) with a small library of reference Pt/C catalysts, all measured under the same conditions. Apart from polycrystalline platinum (Pt-poly) and unsupported Pt-black catalyst, all other catalysts consist of platinum nanoparticles supported on carbon in the form of HGS (BET ≈ 1200 m^2^·g^−1^), Vulcan (BET ≈ 250 m^2^·g^−1^) or high-surface-area carbon black (hereafter denoted as HSA, BET ≈ 800 m^2^·g^−1^) in the case of commercial catalysts. Furthermore, the used catalysts have varying platinum contents as well as different platinum particle sizes and thus provide an overview of the impact of various parameters on the activity properties of standard platinum catalysts (see Table 1).

**Table 1:** The material and activity data of a small library of reference catalysts is summarized and compared to the three model Pt/C materials. TEM images of the reference catalysts with larger magnification are available in Supporting Information File 1 (see Figure S1). The particle diameters of Pt/C 5 nm, Pt/C 3 nm were provided by the manufacturer. The size derived from XRD is in good agreement with the results from TEM. The particle size for Pt-black was calculated from its ECSA, while all other particle sizes were obtained from TEM.

material	manufacturer	particle size [nm]	Pt content [wt %]	electrochemical active surface area [m^2^·g^−1^]	specific activity in HClO_4_/(H_2_SO_4_) [mA·cm^−2^_Pt_]	mass activity in HClO_4_/(H_2_SO_4_) [A·mg^−1^_Pt_]

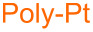	MaTeck	—	100	—	1.8 ± 0.27 (0.43 ± 0.039)	—
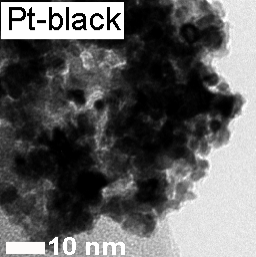	Umicore	10–15	98	18 ± 2	1.4 ± 0.22 (0.27 ± 0.024)	0.26 ± 0.07 (0.049 ± 0.010)
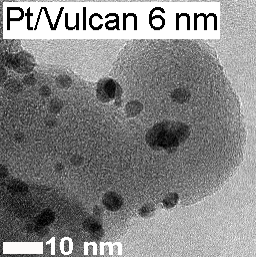	authors’ lab	5–6	21	52 ± 6	0.66 ± 0.10 (0.14 ± 0.013)	0.34 ± 0.09 (0.073 ± 0.015)
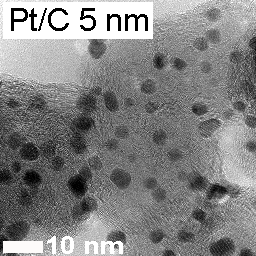	TKK	4.8	51	56 ± 8	0.46 ± 0.07 (0.11 ± 0.012)	0.26 ± 0.08 (0.062 ± 0.014)
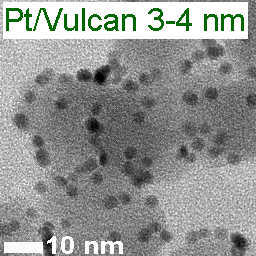	authors’ lab	3–4	20	67 ± 6	0.49 ± 0.06 (—)	0.32 ± 0.07 (—)
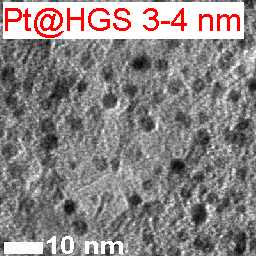	authors’ lab	3–4	20	75 ± 11	0.47 ± 0.07 (0.095 ± 0.009)	0.35 ± 0.09 (0.071 ± 0.017)
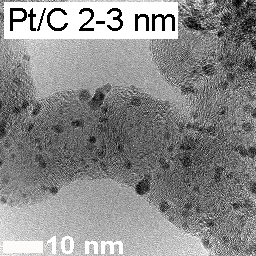	TKK	2.6	46	99 ± 15	0.38 ± 0.06 (0.093 ± 0.008)	0.37 ± 0.11 (0.092 ± 0.022)
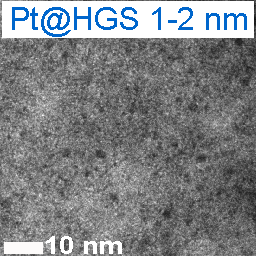	authors’ lab	1–2	20	108 ± 16	0.41 ± 0.06 (0.10 ± 0.009)	0.44 ± 0.13 (0.11 ± 0.026)
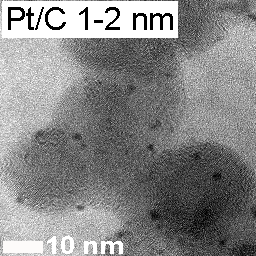	TKK	1–2	10	116 ± 18	0.41 ± 0.06 (0.084 ± 0.008)	0.47 ± 0.14 (0.097 ± 0.024)

**Figure 2:**
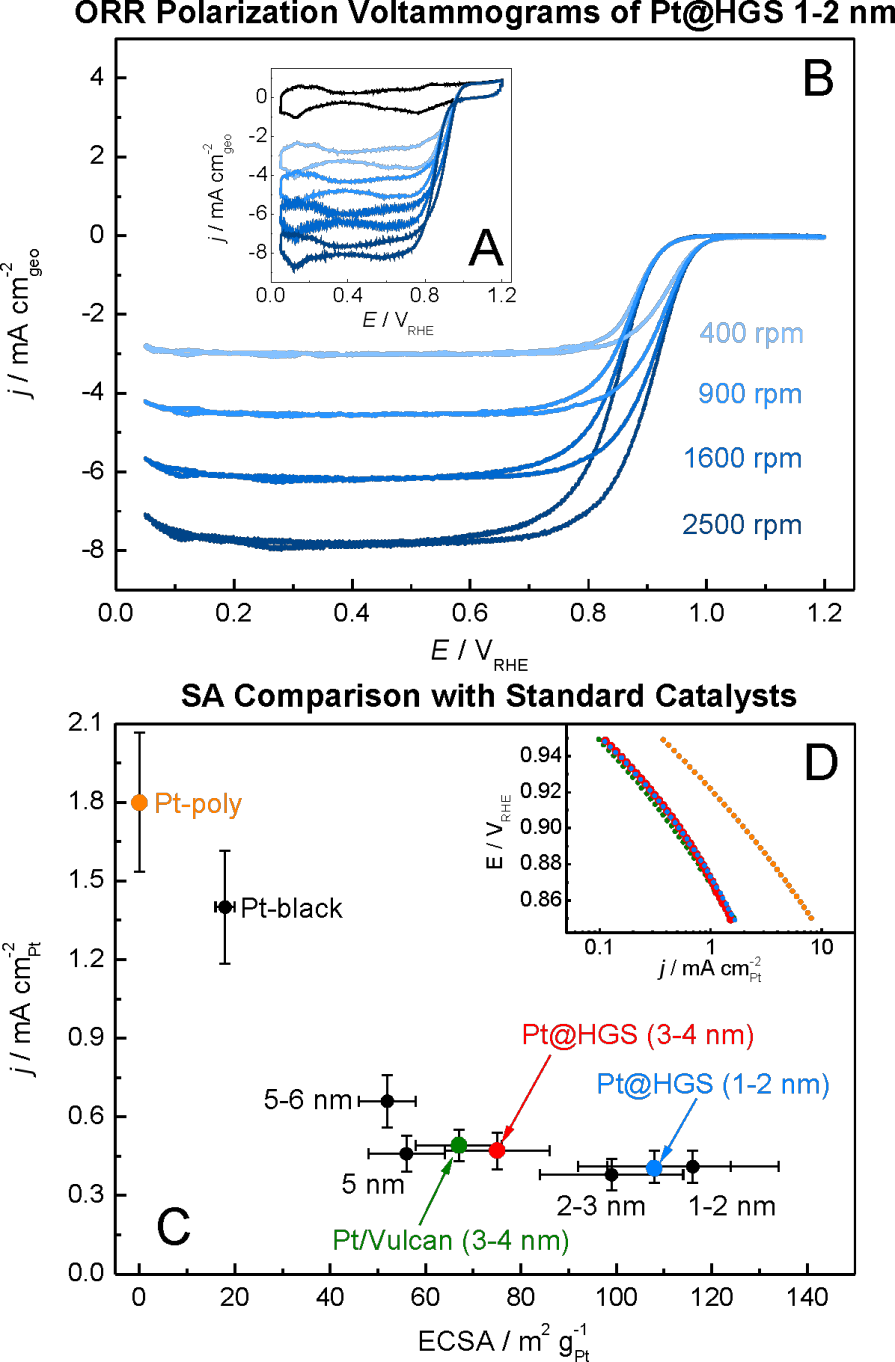
A) ORR cyclic voltammograms of Pt@HGS 1–2 nm in 0.1 M HClO_4_ saturated with Ar (black) and with oxygen at (400–2500 rpm, blue) recorded at room temperature with a sweep rate of 0.05 V·s^−1^. B) Same as in A) after subtraction of capacitive currents. C) Plot of the specific activity as derived from the anodic scan at 0.9 V_RHE_ versus the electrochemically active surface area of platinum illustrating the particle size dependent changes in activity. D) Tafel plots from a representative measurement of Pt-poly (orange), Pt@HGS 1–2 nm (blue), Pt@HGS 3–4 nm (red) and Pt/Vulcan 3–4 nm (green). The three Tafel plots of the Pt/C catalysts are almost identical, which indicates comparable specific activities of Pt@HGS based catalysts with standard high surface area fuel cell catalysts.

The ORR activities in this study were determined by using the so called thin film method [72–73] in a rotating disk electrode (RDE) setup, in two different electrolytes (0.1 M HClO_4_ and 0.1 M H_2_SO_4_). Additionally, compensation for the resistance of the electrolyte by positive feedback has been applied to avoid errors when comparing catalysts with different platinum and carbon content [74]. A representative set of ORR measurements is provided for one of the catalysts studied (Pt@HGS 1–2 nm catalyst) in Figure 2A and Figure 2B. The blue hydrodynamic voltammograms in Figure 2A were recorded in oxygen-saturated 0.1 M HClO_4_ at different rotation rates (400, 900, 1600, 2500 rpm), while the black curve in the same Figure was recorded in argon-saturated electrolyte to estimate the background response. The latter, which remains unaffected by rotation, is subtracted from the curves recorded in oxygen to receive the response entirely due to the ORR (Figure 2B), thus avoiding an overestimation of kinetic currents due to contributions from the capacitive background. The shape of the ORR voltammograms for both Pt@HGS materials are typical for standard platinum catalysts, with the only difference being that the thermal-treated Pt@HGS 3–4 nm material requires electrochemical activation prior to the activity measurement (ca. 200–300 potential cycles between 0.05 and 1.35 V_RHE_ at 0.2 V·s^−1^). The obtained values for the electrochemical active surface area (ECSA), the specific activity (i.e., kinetic current per platinum surface area) as well as the economically relevant mass activity (i.e., kinetic current per mass of platinum) for all catalysts in both electrolytes are all summarized in Table 1, together with the most important material properties.

The specific activity values as a function of the ECSA in 0.1 M HClO_4_ are plotted in Figure 2C (for the according plot in 0.1 M H_2_SO_4_ see Figure S2 in Supporting Information File 1). The unsupported Pt-poly and Pt-black catalysts clearly present a higher specific activity than all supported platinum nanoparticles-based catalysts, and the activity rapidly drops when the particle size decreases down to 5 nm. For the catalysts below this particle size (i.e., in the region 1–5 nm), changes in the specific activity are minor or even within the error of the measurement, which is in line with previous observations [12,14]. The 5–6 nm Pt/Vulcan catalyst exhibits a slightly increased specific activity compared to all other Pt/C catalysts with smaller sizes, however, this may also be an artifact due to the very broad particle size distribution (ranging from 3–13 nm) of this particular catalyst.

The Pt@HGS 1–2 nm and the Pt@HGS 3–4 nm catalysts are characterized by a specific activity comparable with the other catalysts in the particle size range between 1 and 5 nm. This is also evident from Figure 2D, which depicts representative Tafel plots for polycrystalline platinum, Pt/Vulcan 3–4 nm and both Pt@HGS catalysts. The obtained values for the ECSA (see Figure 2C) are in good agreement with the expected values for catalysts of the according nanoparticle diameters, thus indicating that the platinum nanoparticles confined in the mesoporous shell of the HGS are as well accessible as the platinum on other types of carbon supports. As the specific activity does not change significantly even when going to particle sizes as small as 1 nm, the mass activity increases continuously with decreasing particle sizes. The same trends in specific activity, ECSA and mass activity were also confirmed in sulfuric acid (see Table 1 and Figure S2 in Supporting Information File 1).

### Macroscopic stability investigation

While cathode electrocatalysts are rather stable under constant fuel cell operating conditions for hundreds or even thousands of hours, they can degrade rapidly if subjected to more harmful conditions that can for instance occur during start-up and shut-down or in the case of local fuel starvation. Reiser et al. [75] pointed out that potentials at the cathode in this case may locally even exceed values as high as 1.5 V for short periods of time, and already Kinoshita et al. [18] reported that such transitions in potential result in more severe catalyst surface area losses compared to constantly high potentials. These drastic conditions, which can be simulated in electrochemical half-cell experiments by, e.g., subjecting the catalyst to cyclic voltammetry, are a demanding challenge for designing a stable cathode catalyst. Even though it has to be highlighted that the according potential cycling experiments cannot directly reproduce all the phenomena that occur in a real fuel cell as for instance demonstrated recently by Durst et al. [76], they can provide valuable insights about the catalyst performance under well controllable and reproducible conditions.

**Figure 3:**
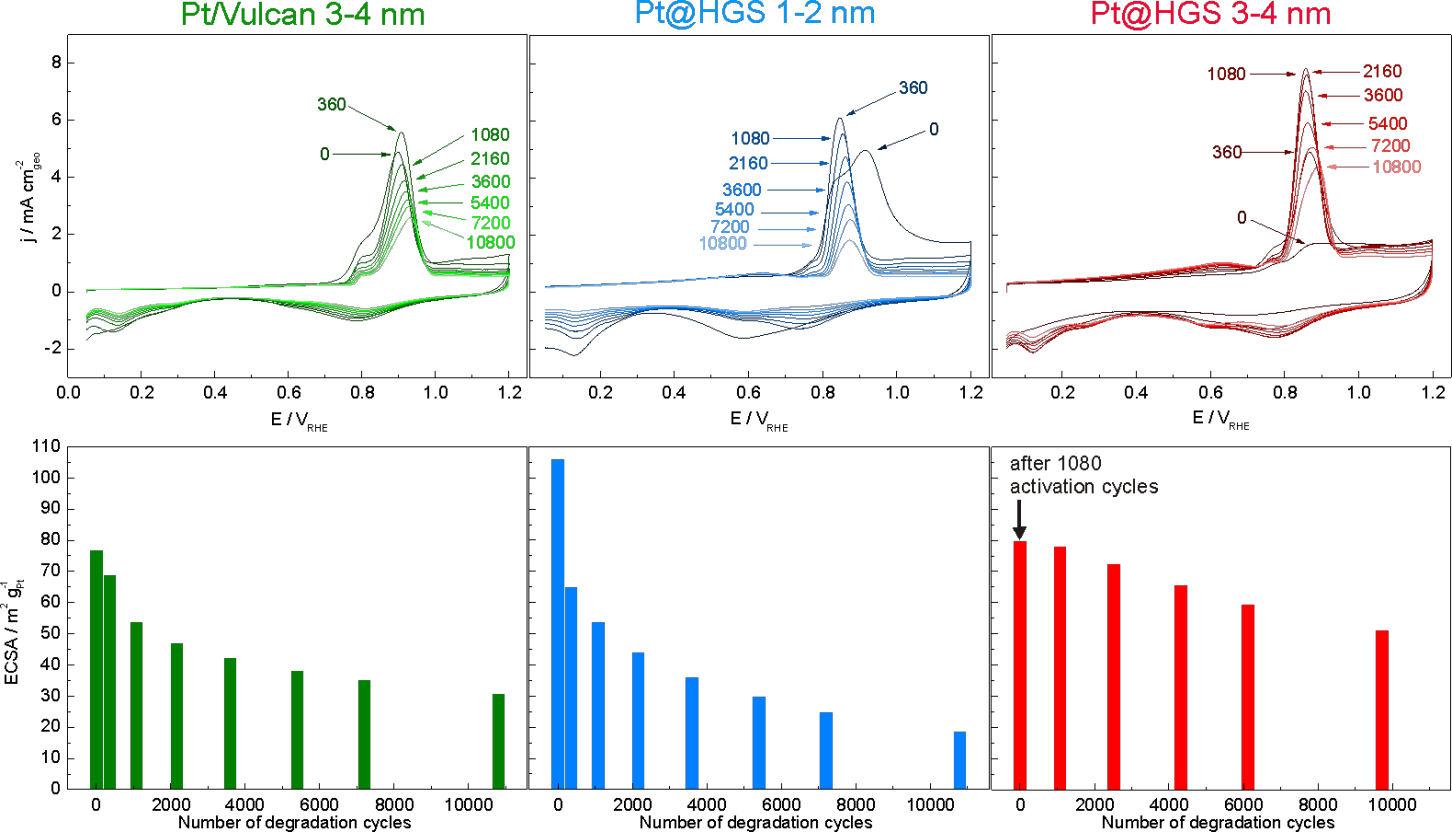
Electrochemical oxidation of a carbon monoxide monolayer (CO-stripping curves) after 0, 360, 1080, 2160, 3600, 5400, 7200 and 10800 degradation cycles for Pt/Vulcan 3–4 nm, Pt@HGS 1–2 nm and Pt@HGS 3–4 nm. The degradation cycles are performed between 0.4 and 1.4 V_RHE_ with a scan rate of 1 V·s^−1^ without rotation at room temperature in argon-saturated 0.1 M HClO_4_ (not shown). CO-stripping voltammograms (top) were recorded between 0.05 and 1.2 V_RHE_ at a scan rate of 0.05 V·s^−1^ to measure the ECSA versus the number of cycles (bottom). The CO-stripping voltammograms of Pt@HGS 3–4 nm are reprinted with permission from [71]. Copyright 2012 American Chemical Society.

According potential changes were thus employed in this work by imposing potential cycles between 0.4 and 1.4 V_RHE_ (1 V·s^−1^, 0.1 M HClO_4_, room temperature) on the three catalyst materials (Figure 3). In this context it has to be emphasized that all three materials, Pt@HGS 1–2 nm, Pt@HGS 3–4 nm and Pt/Vulcan, were tested with an equal amount of platinum on the working electrode, i.e., 30 µg_Pt_·cm^−2^. Namely the amount of catalyst deposited on the electrode can have a significant influence on catalyst stability in thin film degradation tests and thus needs to be considered to enable a fair comparison. The changes in the platinum surface area of the three catalysts were monitored in between the degradation test via electrochemical oxidation of an adsorbed carbon monoxide monolayer, as described in the Experimental section (“Activity measurements”). A typical CO-stripping voltammogram exhibits no current between 0.05 and approximately 0.6 V_RHE_. The features of hydrogen desorption in the hydrogen underpotential deposition (H_UPD_) region, which are typical for cyclic voltammograms of platinum, are not present in that case as the complete platinum surface is covered with adsorbed carbon monoxide, which suppresses the adsorption of hydrogen or other species. The oxidation of the adsorbed CO to CO_2_ finally starts in the positively directed scan at potentials around 0.7 V_RHE_ and results in a pronounced CO-stripping peak. Beyond the CO-stripping peak, the current does not decay to zero, which is due to the formation of platinum oxide on the now CO-free surface. The reduction of the formed platinum oxide in the negatively directed scan (ca. 1.0–0.5 V_RHE_) is followed by the low capacitive currents in the double layer region (ca. 0.5–0.3 V_RHE_) and the adsorption of hydrogen in the H_UPD_ region (ca. 0.3–0.05 V_RHE_). The integration of the area under the CO oxidation signal between the CO-stripping voltammogram and the successive voltammogram in CO-free argon atmosphere (background) is a measure of the active surface area.

According CO-stripping experiments were carried out after 0, 360, 1080, 2160, 3600, 5400, 7200 and 10800 degradation cycles. A representative measurement for each of the three catalysts is depicted in Figure 3. For the Pt/Vulcan catalyst, the most rapid decrease in ECSA occurs at the beginning of the degradation test and, in particular, almost half of the total losses in ECSA take place in the first thousand degradation cycles, which corresponds to a tenth of the total amount of imposed potential cycles on the catalyst. After 10800 degradation cycles, the remaining ECSA is 30 m^2^·g^−1^ compared to initially 77 m^2^·g^−1^, corresponding to a loss of about 61% of the original ECSA.

The ECSA loss is more severe for the Pt@HGS 1–2 nm catalyst. The smaller particle size is reflected in the higher initial ECSA of 106 m^2^·g^−1^. However, already after the first 360 degradation cycles, the ECSA of the Pt@HGS 1–2 nm catalyst falls below the ECSA value of the Pt/Vulcan 3–4 nm catalyst. After 10800 degradation cycles, only 18 m^2^·g^−1^ are left, i.e. the total loss of ECSA of the Pt@HGS 1–2 nm catalyst corresponds to 83%. The high initial loss of ECSA for this material, already within the first 360 degradation cycles, is accompanied with a characteristic change of the shape of the carbon monoxide oxidation peak. In particular, before the degradation cycles, the oxidation peak spans a very broad potential range from 0.72 V_RHE_ to potentials above 1.0 V_RHE_, and has a pronounced shoulder at about 0.85 V_RHE_ and a maximum at 0.91 V_RHE_. After 360 degradation cycles, the peak has become sharper, the maximum shifts to a more negative potential (0.85 V_RHE_), while no significant carbon monoxide oxidation is observed anymore above 0.91 V_RHE_. Since the peak potential of the CO oxidation is more positive for smaller particles [77–79], the described changes of shape and the peak potential shift indicate that the CO oxidation before the treatment takes place mainly on small nanoparticles, which are not present anymore after the first 360 degradation cycles. Thus, the initial drastic surface area loss is most likely linked to a loss/rearrangement of the smallest platinum nanoparticles of the Pt@HGS 1–2 nm catalyst. Moreover, the increase of the current and the evolution of a maximum in the CO-stripping current after 360 degradation cycles at 0.85 V_RHE_, at which a peak shoulder was observed before degradation, can be interpreted as an indication for an increase of the amount of larger particles. This analysis is also confirmed by the shift of the oxide reduction peak toward more positive potentials after 360 degradation cycles, which is characteristic for the reduction of platinum oxide formed at less oxophilic, larger particles [11].

To confirm that these observations are not related to the support, we imposed the same degradation protocol on a commercial Pt/C catalyst with an average platinum particle size of 1–2 nm (TKK, 10 wt % platinum, see also Table 1), and the same characteristic shape changes in the CO-stripping voltammograms were observed (see supporting Figure S3). Thus, the above mentioned observations are solely due to the initially present small platinum particles, which are not stable even if subjected to only a few degradation cycles, leading to rapid ECSA losses for both the Pt@HGS and the commercial Pt/C catalysts of 1-2 nm size.

A different picture evolves for the Pt@HGS 3–4 nm catalyst, for which no strong CO oxidation, but also no pronounced platinum features are observed before the degradation cycles. This indicates that platinum is initially not accessible to the electrolyte, and that the first potential cycles are necessary for the “activation” of the catalyst material, which is reflected in the increase of both the CO-stripping signal, as well as the characteristic platinum features in the H_UPD_ and platinum oxide regions. This activation process is initially fast, so that about 90% of the catalyst surface is accessible already after less than 500 potential cycles, while afterwards the ECSA increases slowly and reaches its maximum value only after 1080 potential cycles. The need for this harsh activation is attributed to the cleaning of the platinum from carbon impurities that are introduced upon thermal treatment during the material synthesis. The accessibility of the platinum particles in the network is limited by pores that might be blocked by the sintered platinum nanoparticles, as the average particle diameter after the thermal treatment is in the range of the pore size distribution in the mesoporous network. Potential cycling may help to corrode the carbon that is in contact with the sintered platinum particles allowing the recovery of accessibility. The ECSA obtained after 1080 activation/degradation cycles is 80 m^2^·g^−1^, which is comparable to the initial ECSA of the Pt/Vulcan 3–4 nm catalyst and in agreement with what is expected for a catalyst of the according platinum particle size. Throughout the remaining 9720 degradation cycles, the ECSA decays with a lower rate compared to the other two catalysts. The final ECSA of the Pt@HGS 3–4 nm material is 51 m^2^·g^−1^, i.e., the total loss after 9720 cycles is less than 36%, which is much lower than the 83% for the Pt@HGS 1–2 nm catalyst. Even more important, the Pt@HGS 3–4 nm catalyst maintains a significantly higher absolute ECSA than the Pt/Vulcan 3–4 nm catalyst (30 m^2^·g^−1^) after the same number of cycles, which would correspond to a higher voltage and power output in fuel cell operation over an extended time.

Interestingly, the sharp initial decay of the surface area is completely absent for the Pt@HGS 3–4 nm catalyst, while it is quite severe for the other two materials. The reasons for this have not been completely resolved so far, but two likely contributions shall be mentioned here. Shao-Horn et al. [15] suggested, that this fast initial degradation may be explained by the loss of the smallest platinum particles as a result of a rapid dissolution. This suggestion is in agreement with our observations for the Pt@HGS 1–2 nm catalyst, which shows very high initial degradation rates, due to a massive loss of the smallest particles. In contrast, the coalescence of particles being initially in contact can also lead to fast initial degradation for many materials even with larger particle size. This would explain the behavior of the Pt/Vulcan 3–4 nm catalyst and might be important for comparable fuel cell catalysts with high platinum loadings on carbon supports with low surface areas. At a later stage in the degradation process, when particles in close vicinity to each other have already merged into larger ones or when the distances between the platinum particles have increased also due to other degradation mechanisms, further agglomeration and coalescence becomes less likely. The overall degradation becomes less severe since effectively only dissolution remains. These changes in the individual contributions of certain degradation pathways (particle detachment and coalescence) to the overall surface area loss over time seem to be absent in the case of the Pt@HGS 3–4 nm. Thus the stability is only limited by the dissolution of Pt particles from the beginning on, which is in general hard to circumvent. A more detailed microstructural analysis, as presented in the next section is, therefore, necessary to support this hypothesis.

### Stability investigation on the nanoscale

**Figure 4:**
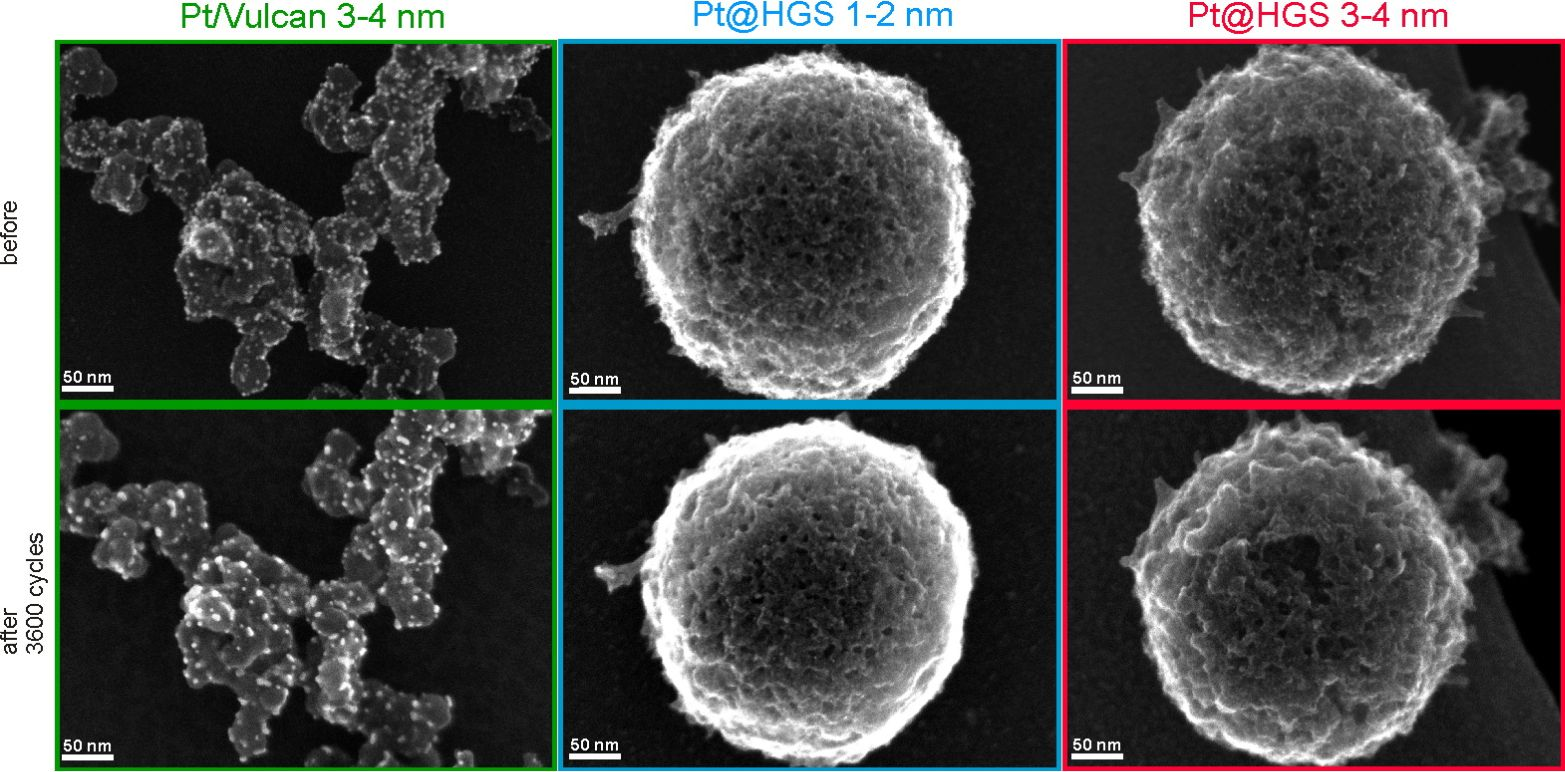
IL-SEM of Pt/Vulcan 3–4 nm (green), Pt@HGS 1–2 nm (blue) and Pt@HGS 3–4 nm (red) after 0 (top) and after 3600 (bottom) potential cycles between 0.4 and 1.4 V_RHE_ in 0.1 M HClO_4_ at a scan rate of 1 V·s^−1^. IL-SEM visualizes the surface morphology of the materials, in particular the support structure, which is demonstrated not to undergo significant changes during potential cycling at room temperature.

The above described tests focused on the macroscopic differences in loss of ECSA for catalysts with different material properties. To gain insight into the underlying reasons for the different stability characteristics and thus into the impact of the material design on the degradation pathways, investigations at the nanoscale -level are explored in the same manner as described in [71]. IL-SEM was used to study morphological and structural aspects of the support for the Pt/Vulcan 3–4 nm, Pt@HGS 1–2 nm and Pt@HGS 3–4 nm catalysts before and after 3600 potential cycles between 0.4 and 1.4 V_RHE_ (scan rate 1 V·s^−1^, room temperature) in 0.1 M HClO_4_. Three representative catalyst locations are shown in Figure 4, with the catalysts before and after electrochemical treatment always on top and below, respectively.

The dissimilarity of the two types of employed carbon supports, Vulcan and HGS, becomes evident from Figure 4. While Vulcan is made of primary carbon particles of 10–40 nm that form aggregates with the typical branched chain-like structure of carbon black materials, HGS are characterized by their ball-shaped morphology. The interior of the latter is hollow and thus every sphere contains one large macropore, while the approximately 50 nm thick shell – where the platinum nanoparticles are confined – consists of a three-dimensional interconnected mesoporous network with two maxima in the pore size distribution around 4 and 10 nm [71]. When comparing the untreated and the electrochemically treated catalyst locations in Figure 4, the major observation is that no significant changes take place, neither in the morphology nor in the external surface topology of any of the three samples under investigation. Thus, a structural breakdown of the carbon support due to strong carbon corrosion is not a dominant degradation mechanism for the three materials under these conditions. This is in good agreement with previous IL-TEM studies [40], in which indications for massive carbon corrosion have been found only rarely and at a few catalyst locations at room temperature, while structural breakdown of the carbon support is to be expected mainly at elevated temperatures [49,54]. Even if a loss of structural integrity of the support is not observed, carbon corrosion especially in proximity to platinum cannot be excluded.

**Figure 5:**
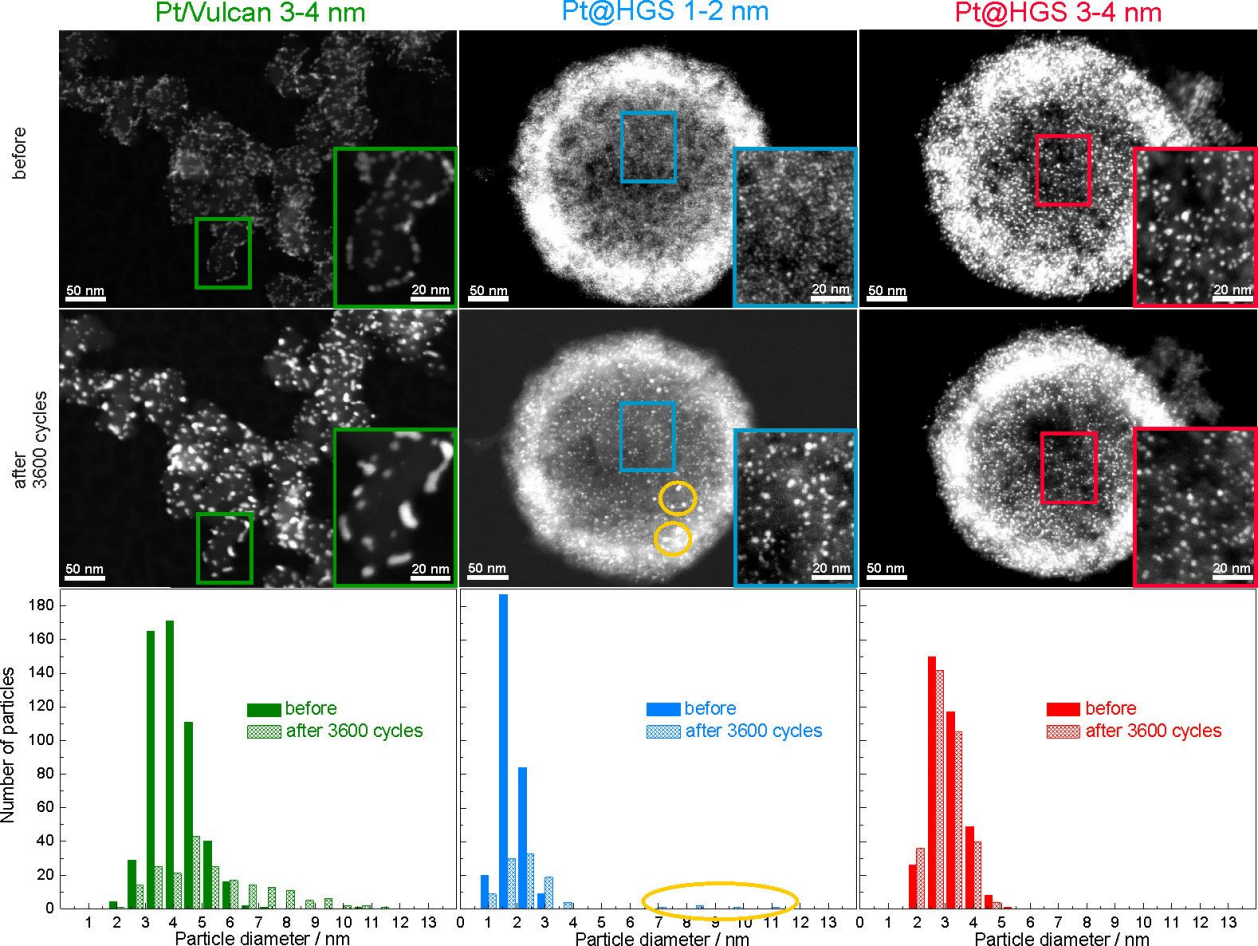
Identical location dark field IL-STEM of Pt/Vulcan 3–4 nm (green), Pt@HGS 1–2 nm (blue) and Pt@HGS 3–4 nm (red) after 0 (top) and after 3600 (middle) potential cycles between 0.4 and 1.4 V_RHE_ in 0.1 M HClO_4_ at a scan rate of 1 V·s^−1^ at room temperature. The insets highlight sub-regions in the micrograph. The yellow circles for the Pt@HGS 1–2 nm material mark several particles which result from untypical, strong particle growth. The change in particle size distribution for all three Pt/C materials is additionally depicted at the bottom. Bars referring to the same particle diameter before (filled) and after degradation (shaded) are pictured next to each other.

While an identification of the platinum nanoparticles on the rough and porous carbon surface of the two Pt@HGS samples via SEM at this magnification is not straightforward, the platinum particles can be readily identified on the Pt/Vulcan 3–4 nm material. Most remarkably, a clear increase in the average particle size for the Pt/Vulcan is already visible from the SEM micrographs. A still better visualization of the platinum nanoparticles can be achieved with the higher resolution in TEM, where a 2D projection of the complete object under investigation discloses also the platinum nanoparticles inside and on the back side of the porous network. Figure 5 shows dark field scanning transmission electron microscopy (DF-STEM) images for all three materials at the same locations as in Figure 4, before (top) and after (middle) the above described electrochemical treatment. A first glance at the three materials before electrochemical treatment indicates that for all of them the platinum particles are well dispersed over the complete carbon support. The impression of the high particle density in the Pt@HGS materials originates from the particles being located at different “levels” throughout the 50 nm thick shell, i.e., some of the particles that appear to be close to each other are tens or hundreds of nanometers (if located at the other side of the sphere) apart. The dispersion of platinum is especially fine for the Pt@HGS 1–2 nm catalyst, as reflected in the blue inset and in the particle size distribution before the degradation test. The Pt@HGS 3–4 nm material (which is produced from the Pt@HGS 1–2 nm material through a thermal-treatment step up to 900 °C for several hours in an inert atmosphere) has clearly undergone a mild sintering of the smaller platinum nanoparticles that led to an average particle size of about 3–4 nm, as can be seen in the red inset and the according particle size distribution before the electrochemical treatment. The particle growth due to the thermal treatment is remarkably well-controlled and the resulting particle size distribution is very monodisperse compared to what is known for other Pt/C materials that have undergone a comparable treatment. This well-defined particle growth is attributed to the defined pore size distribution within the mesoporous network. The comparison between the DF-STEM images before and after degradation shown in Figure 5 provides various insights into the degradation behavior of the three catalysts, which is summarized below.

### Description and interpretation of Pt/Vulcan 3–4 nm degradation

**Figure 6:**
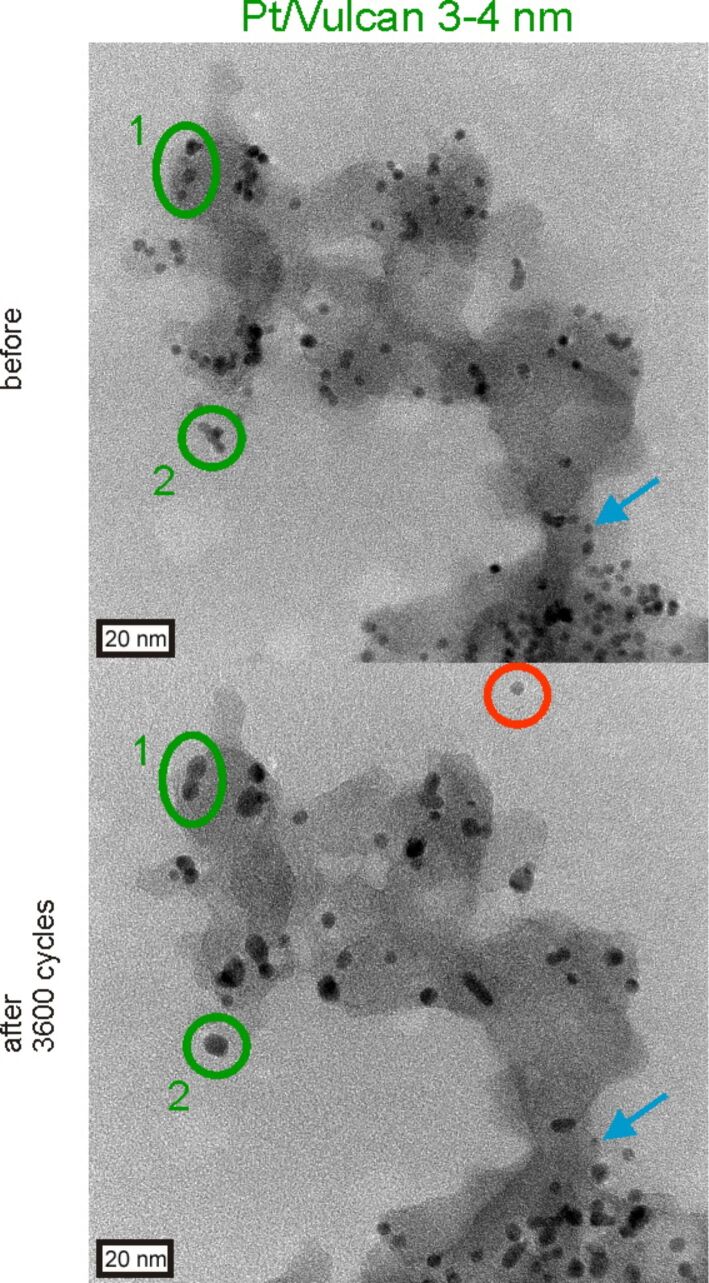
IL-TEM micrographs of Pt/Vulcan 3–4 nm after 0 and after 3600 potential cycles between 0.4 and 1.4 V_RHE_ in 0.1 M HClO_4_ (scan rate 1 V·s^−1^, room temperature). Blue, red and green symbols mark dissolving, detached and coalescing platinum particles.

A significant alteration of the Pt/Vulcan 3–4 nm catalyst is shown in Figure 5, with a decrease in the total amount of platinum particles and an increase in the average platinum particle size. The strong particle growth is the most obvious occurring degradation process and the shape of the formed clusters in the insets with higher magnification indicates coalescence to be an important degradation mechanism. The particle growth is also clearly reflected in the change in particle size distribution with a tailing towards larger particle sizes, which is frequently interpreted as evidence for agglomeration and coalescence in post-mortem TEM investigations of fuel cell catalysts. However, particle growth is not the only pathway responsible for the loss of platinum ECSA for the Pt/Vulcan 3–4 nm catalyst. Figure 6 is a standard IL-TEM image of another location of Pt/Vulcan 3–4 nm, which reveals that several degradation processes are taking place in parallel. Particle growth is accompanied by detachment of particles from the Vulcan support, likely as a consequence of the corrosion of carbon in direct contact with platinum (red circle), and dissolution of platinum particles (blue arrow).

**Figure 7:**
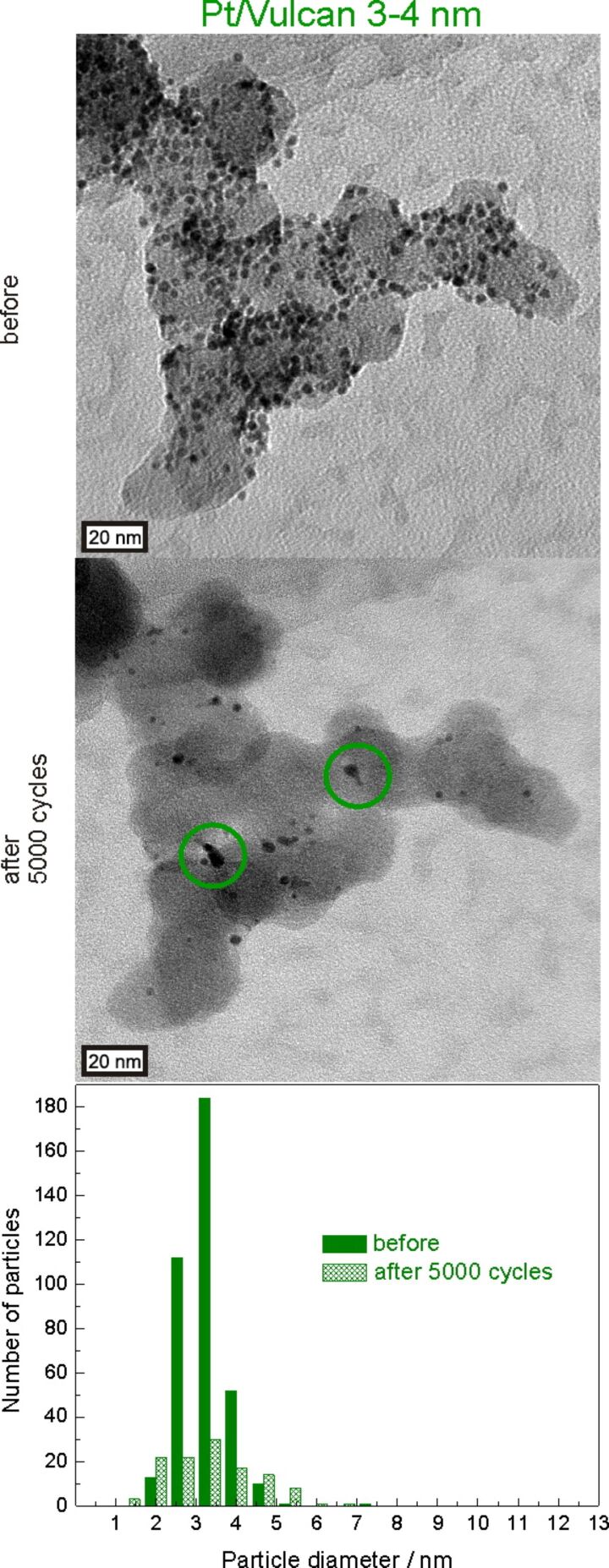
IL-TEM micrographs of the Pt/Vulcan 3–4 nm catalyst before and after 5000 potential cycles between 0.4 and 1.4 V_RHE_ in 0.1 M HClO_4_ (scan rate 1 V·s^−1^, room temperature). Green circles indicate examples for agglomeration of platinum nanoparticles. The particle size distributions before and after 5000 degradation cycles indicate both particle growth and dissolution to occur. Bars referring to the same particle diameter before (filled) and after degradation (shaded) are pictured next to each other.

Coalescence can originate from particles that migrate on the carbon support during the degradation test, collide and ”melt” together when they come into contact, as pointed out by the 1st green circle where “necking” (i.e. a thin bridge between two particles) can be identified. We hereafter refer to this type of particle growth mechanism as agglomeration due to migration, as it is often found in the literature [15]. The situation is slightly different for the platinum particles depicted by the 2nd green circle, where the particles are already in contact with each other from the very beginning and coalescence is immediately possible resulting in larger particles with reduced surface area. We hereafter refer to this type of mechanism as *coalescence due to initial contact*. In this case, coalescence is most likely controlled by surface diffusion processes of platinum atoms of the touching particles. This mechanism seems to be the dominant process also in Figure 5, in which a large number of particles are close to each other already before the degradation test, despite the comparatively good dispersion of particles. To investigate the contribution of the initially dominating coalescence to the overall surface area loss at a different stage we also recorded IL-TEM micrographs on Pt/Vulcan 3–4 nm after 5000 degradation cycles (between 0.4 and 1.4 V_RHE_, scan rate 1 V·s^−1^, 0.1 M HClO_4_, room temperature), as shown in Figure 7.

Naturally the changes in microstructure are more severe at this later stage in the degradation process and the increase in inter-particle distances becomes even more obvious than after 3600 cycles. As can be seen from the representative catalyst location depicted in Figure 7 and based on the shape of the formed clusters, traces of coalescence can still be observed. However, while after 3600 degradation cycles (see Figure 5 and Figure 6) particle growth due to coalescence appears to play the prevailing role and the average particle size increases strongly, no further increase in average particle size is observed when the degradation test is performed for a total of 5000 degradation cycles. In fact, a large number of platinum particles decrease in size due to dissolution, which appears to be the more important degradation pathway regarding its relative contribution to surface area loss at this later stage in the degradation process. Moreover, many clusters that were formed via coalescence are small compared to what would be expected on the basis of the amount of platinum particles initially present in that region, which indicates that also the formed clusters shrink as a consequence of dissolution. These observations are also reflected in the comparison of particle size distributions before and after 5000 degradation cycles. While the number of medium sized particles has decreased, both the amount of larger and smaller particles has increased, confirming that not only coalescence, but also dissolution of platinum is taking place.

It needs to be emphasized that considering the presence of dissolution it cannot be excluded that Ostwald ripening may contribute to the observed particle growth. Namely, in a real fuel cell electrode dissolved platinum ions could either precipitate in the ionomer or on larger particles within the extended 3D structure of the catalyst layer, besides being washed out with the exhaust water. In an IL-TEM experiment, however, only a very small quantity of electrocatalyst is deposited on the TEM grid and thus no extended 3D catalyst layer is present. The catalyst on the grid is exposed to a large volume of electrolyte and thus the concentration of dissolved platinum species remains low, which makes platinum re-deposition less likely to occur. Only for cases in which high amounts of platinum dissolve in a very short period of time, the dissolved platinum concentration at the interface may be sufficient to observe re-deposition and Ostwald ripening in an IL-TEM experiment. The importance of the 3D structure of the catalyst layer for the observation of significant re-deposition as highlighted previously [16,40], was recently confirmed by the investigation of catalyst layers with thicknesses of several micrometers by using IL-SEM [64].

### Description and interpretation of Pt@HGS 1–2 nm degradation

As already seen from the macroscopic stability test, the Pt@HGS 1–2 nm catalyst is the least stable of the three catalyst materials, which was attributed to a massive loss of the smallest platinum particles in the initial degradation stage based on an analysis of the CO-stripping features. An unambiguous identification of the underlying degradation mechanisms for the Pt@HGS 1–2 nm material cannot be easily accomplished, because of the complex structure of the HGS support and the small particle size. Nevertheless, the IL-STEM picture in Figure 5 confirms this picture, as a very large amount of the smallest platinum particles is not present anymore after 3600 degradation cycles, while the overall particle density is decreased. The drastic loss of small particles is furthermore reflected in the particle size distribution, in which more than 60% of the particles are gone after the accelerated-aging test and the average particle size has moderately increased from 1.8 nm to 2.5 nm. Indeed platinum dissolution (possibly with successive re-deposition of some of the dissolved platinum) appears as the most likely cause for the strong ECSA losses of the Pt@HGS 1–2 nm material.

In addition, the formation of large platinum particles with a size of about 10 nm highlights a minor contribution of particle growth to the overall degradation. As major dissolution is observed under these conditions in conjunction with the particle growth, a dissolution/re-deposition mechanism may occur. However, coalescence is an at least as likely contributor to the observed particle growth. In particular, the fact that the Pt@HGS 3–4 nm catalyst is prepared by sintering upon thermal treatment of the Pt@HGS 1–2 nm catalyst, shows that coalescence is feasible and that enough platinum nanoparticles are initially in sufficient proximity to each other to allow agglomeration and coalescence. Moreover, coalescence can take place for the Pt/Vulcan 3–4 nm material under exactly the same aging conditions. Therefore it is reasonable to assume that it also plays an important role in the particle growth observed for the Pt@HGS 1–2 nm material.

### Description and interpretation of Pt@HGS 3–4 nm degradation

As shown already in the “macroscopic stability test”, the Pt@HGS 3–4 nm catalyst is the most stable of the three investigated Pt/C materials, as it is able to preserve a high ECSA throughout the whole degradation test. The high stability of this catalyst under the aggressive potential cycling treatment is confirmed via IL-STEM in Figure 5, which indicates that there are no significant changes in the catalyst material after 3600 cycles and that the changes in the particle size distribution are less significant compared to the other two materials. Only a very slight increase in the number of the smallest particles and a concomitant minor decrease in the number of larger particles can be interpreted as an indication of dissolution.

**Figure 8:**
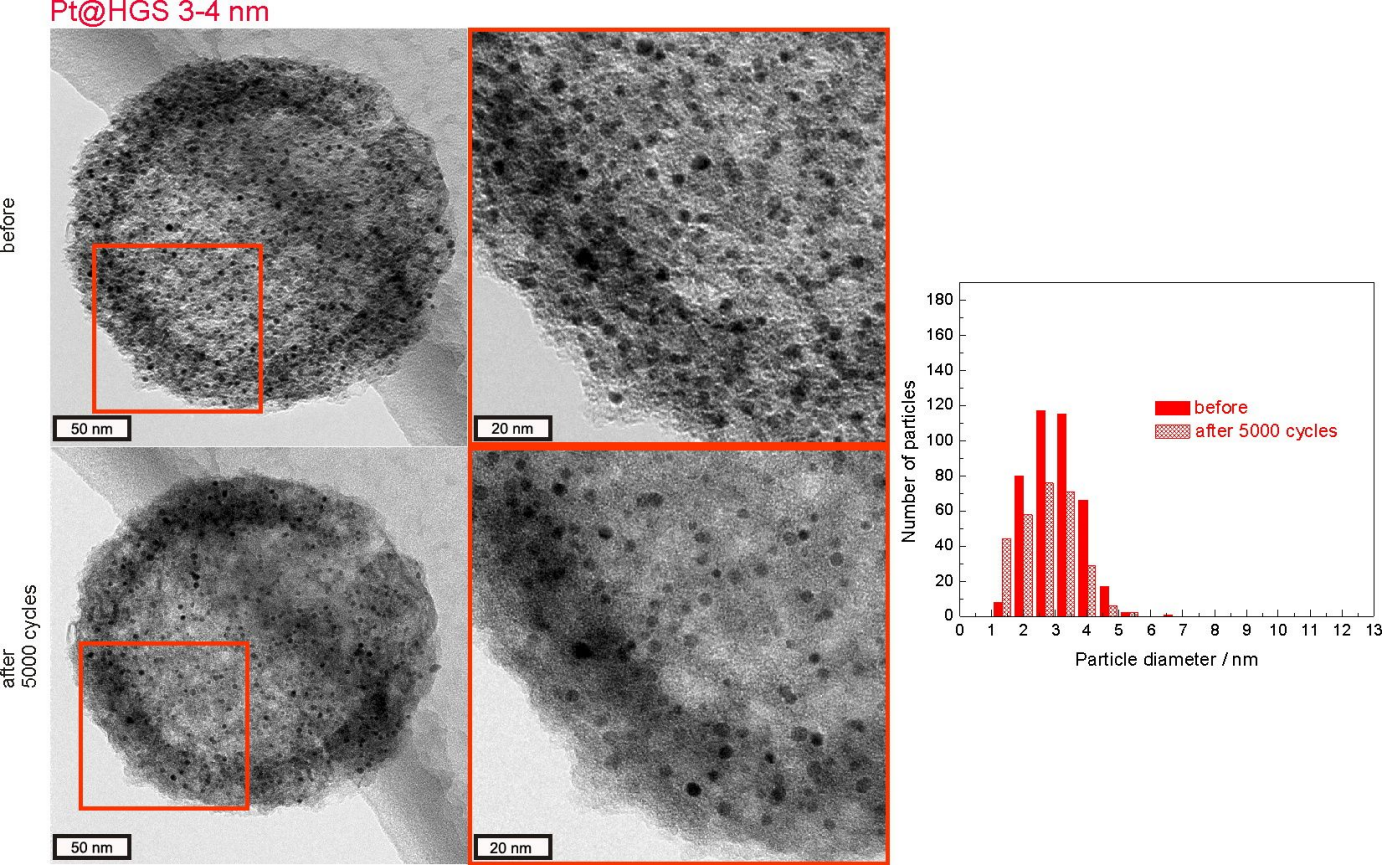
IL-TEM micrographs of the Pt@HGS 3–4 nm catalyst before and after 5000 potential cycles between 0.4 and 1.4 V_RHE_ in 0.1 M HClO_4_ (scan rate 1 V·s^−1^, room temperature). The red rectangles in the micrographs mark regions, which are magnified on the right. Filled bars refer to the particle size before, shaded bars to the particle size after degradation.

In this context, it is important to emphasize that the Pt@HGS 3–4 nm catalyst requires several hundreds of activation cycles, and therefore Figure 5 depicts a somewhat favorable situation for the Pt@HGS 3–4 nm material. Therefore, we additionally performed IL-TEM before and after 5000 degradation cycles (Figure 8) instead of 3600 cycles only, to take the necessary activation amply into account. The comparison of the degradation observed after 5000 cycles for the Pt@HGS 3–4 nm material with the other two materials after 3600 cycles reveals that the Pt@HGS 3–4 nm material is still significantly less degraded, which confirms the findings of the “macroscopic stability tests”. The particle size distribution before and after 5000 cycles depicts more clearly now that the number of small particles increases while the number of larger particles decreases, which is evidence for platinum dissolution to occur. It also cannot be excluded that the detachment of platinum particles from the external surface of the HGS catalyst, which are not incorporated in the mesoporous network, may contribute to the modest decrease in total number of platinum particles. While the Pt/Vulcan catalyst of the same particle size shows particle growth and particularly coalescence as a dominant process, no particle growth is observed for the Pt@HGS 3–4 nm catalyst under the applied conditions. Even though a few platinum particles appear to move slightly in the mesoporous network, the majority of particles can be identified separated one by one after the aging test (see Figure S4 in Supporting Information File 1). Thus also the IL-measurements confirm the improved stability behavior of Pt@HGS 3–4 nm. It is worth to mention in this context that first measurements in real fuel cells also indicate an improved stability in line with these findings [71].

### Design considerations

The results presented so far, can be summarized in the following. A structural breakdown of the carbon support is not playing a dominant role at room temperature for the three investigated catalysts. Nevertheless, the importance of such a mechanism may strongly increase with increasing temperature [54]. Platinum dissolution is an important degradation mechanism for all three investigated catalysts. The massive loss of smallest particles for the Pt@HGS 1–2 nm catalyst indicates that dissolution is most severe for materials with very small platinum particle size. Pt/Vulcan 3–4 nm and to some extent Pt@HGS 1–2 nm suffer from particle growth, which is mainly attributed to coalescence especially at the initial stage of the degradation process. In contrast, Pt@HGS 3–4 nm does not show any significant particle growth.

Despite some similarities, there are distinct differences in the degradation behavior of the three materials. In order to understand how materials can be designed to mitigate degradation mechanisms and still maintain high ORR activities, it is important to analyze which properties are responsible for the observed differences in the degradation behavior. In the following, we will use the activity data presented in Table 1 and Figure 2 along with the degradation data for the herein investigated catalysts, as well as data from catalysts from other IL-TEM studies, to evaluate the effect of material properties on the activity and degradation behavior of Pt/C catalysts.

### Effect of inter-particle distance

**Figure 9:**
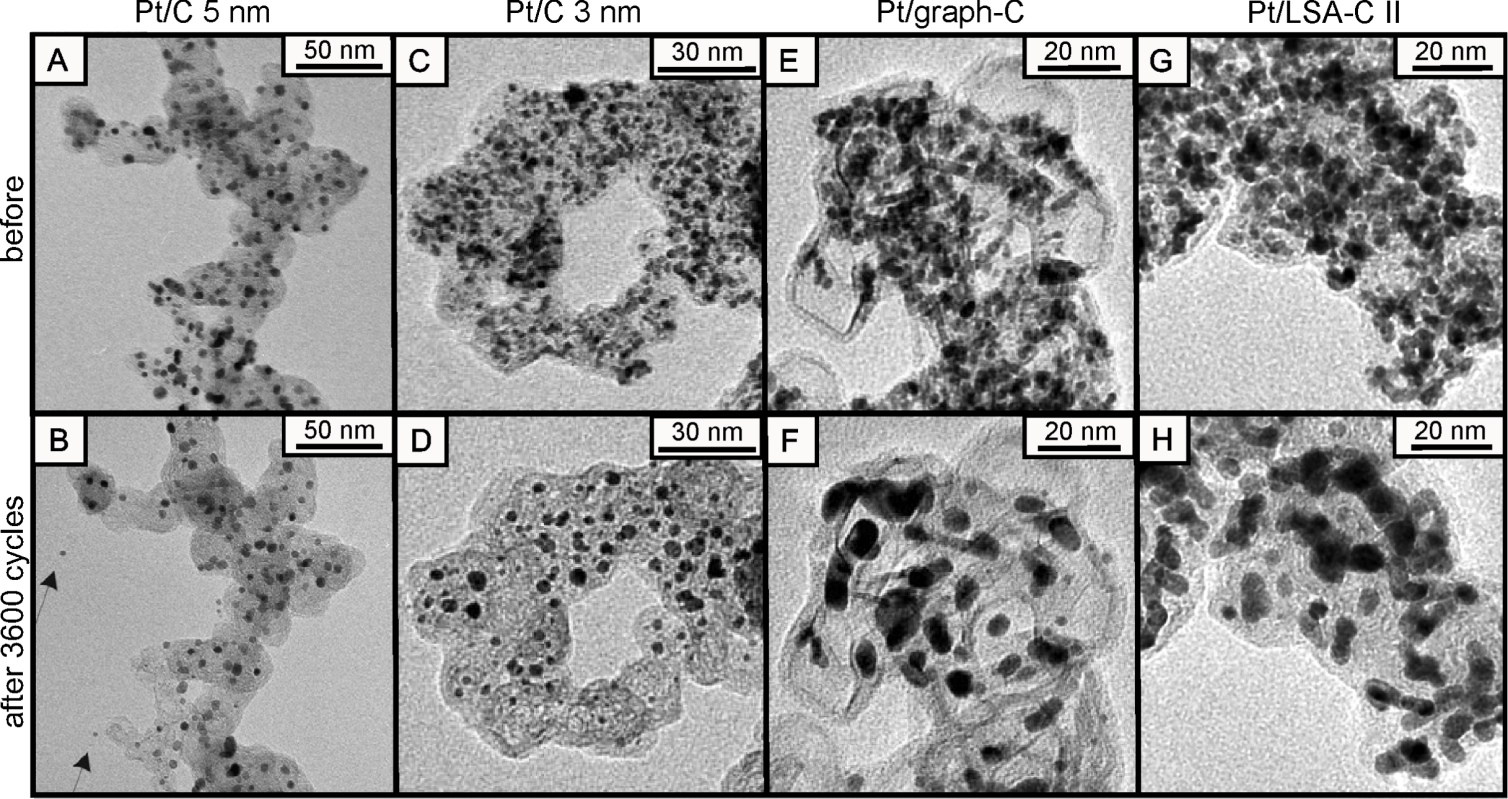
IL-TEM micrographs from degradation studies on four Pt/C fuel cell catalysts. Pt/C 5 nm (A,B) and Pt/C 3nm (C,D) are the same catalysts as depicted in Table 1 and Figure 2. Pt/graph-C (E,F) is a catalyst with graphitized carbon support and Pt/LSA-C II (G,H) is a catalyst with a (transition-metal modified) low surface area support. More information about these and further catalysts can be found in Table 2. A), C), E) and G) depict the catalysts before potential cycling, while B), D), F) and H) are micrographs of the identical locations after 3600 potential cycles between 0.4 and 1.4 V_RHE_ (scan rate 1 V·s^−1^; 0.1 M HClO_4_, room temperature). A) and B) were reprinted from [52] with permission; Copyright 2008 Elsevier. C)–F) were reproduced from [67] with permission; Copyright 2012 The Electrochemical Society. G) and H) were kindly provided by Arenz and co-workers.

Particles can agglomerate due to migration on or shrinkage of the carbon support. When particles establish contact or are already in contact after synthesis, coalescence due to surface diffusion of platinum atoms during potential cycling leads to successive decrease in surface area. The probability of agglomeration and coalescence increases with decreasing distance between particles on the support, since shorter travelling is required to establish contact. As degradation proceeds, the distance between the particles will increase and the contribution of agglomeration and coalescence in the overall surface area loss should decrease over time. Agglomeration and in particular coalescence due to initial contact are not only of importance for the homemade Pt/Vulcan 3–4 nm material, but also for many commercial catalysts. Figure 9 summarizes IL-TEM data from three different studies [52,66–67] for four different Pt/C fuel cell catalysts, which were subjected to the same accelerated-aging test as the three Pt/C catalysts in this study (0.4–1.4 V_RHE_, 1 V·s^−1^, 0.1 M HClO_4_, room temperature). More details about the characteristics of these Pt/C materials (as well as others that will be described later) are available in Table 2 (see below). While no agglomeration and coalescence is seen for the Pt/C 5 nm catalyst, particle growth is present for the Pt/C 3 nm catalyst, and even stronger particle growth can be found for the Pt/LSA-C II and Pt/graph-C materials. Pt/LSA-C II is described as a catalyst with a transition-metal modified low surface area (LSA) carbon support, while Pt/graph-C is a catalyst with a graphitized carbon support. Indeed, it can be qualitatively seen in the IL-TEM micrographs (Figure 9) that the largest initial inter-particle distances are observed for the Pt/C 5 nm, followed by the Pt/C 3 nm catalyst, and only very small inter-particle distances are present for the Pt/LSA-C II and the Pt/graph-C materials. In fact, for the latter two catalysts, a large fraction of platinum nanoparticles are in contact already from the beginning and thus coalescence due to initial contact appears to be the likely cause for the massive particle growth of these materials.

It needs to be noted that highly graphitized supports are often used to prevent carbon corrosion however such supports quite commonly exhibit small specific surface areas. At the same time, high platinum loadings are often desirable to reduce the thickness of the catalyst layer in the fuel cell, and thus to reduce the mass transport limitations in the catalyst layer. The combination of the two – high platinum loadings and small carbon surface areas – inevitably leads to Pt/C catalysts with many platinum particles in close proximity or even in contact to each other. Both Pt/LSA-C II and Pt/graph-C are good examples in this respect. While such materials appear susceptible towards agglomeration and coalescence, they do show high carbon corrosion tolerance.

The determination of the inter-particle distance along the surface of the support is not straightforward, as TEM only provides a 2D projection of a 3D reality. Thus a rough estimation of the inter-particle distance of various Pt/C catalysts can only be achieved by taking certain material properties into account, i.e., (i) the platinum content (ii) the surface area of the support (iii) the platinum particle size and (iv) the homogeneity of the distribution of the platinum nanoparticles. Assuming a two-fold monodisperse Pt/C catalyst, i.e., a Pt/C catalyst consisting of both platinum nanoparticles of the same diameter and a perfectly equidistant distribution of those particles on the carbon support, then an equation can be derived from pure geometric considerations to estimate the “average inter-particle distance”, *l*, as a function of the above mentioned parameters (Equation 1):

[1]
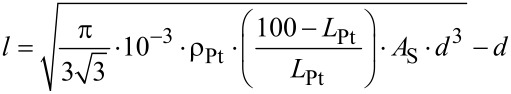


Where *l* is the “average inter-particle distance” (nm), ρ_Pt_ is the density of platinum (21.45 g·cm^−3^), *L*_Pt_ is the platinum content (wt %), *A*_s_ is the specific surface area of the support (m^2^·g^−1^), and *d* is the platinum particle diameter (nm). The derivation and a more detailed explanation of the equation are provided in Supporting Information File 1. It should be noted that a similar equation was derived by Watanabe et al. [80] to study the impact of inter-particle distances on catalyst activity, however, Equation 1, which is provided here to study the probability of agglomeration and coalescence, additionally considers the extension of the platinum particles (i.e., the inter-particle distance is estimated between the surfaces of the particles rather than between their centers).

The average inter-particle distance (AID) corresponds to the length a particle has to travel along the support surface to meet the next platinum particle. Of course, the AID has to be understood as an indicative average value, for the following reasons: (i) since a two-dimensional ideally homogeneous distribution was assumed, the heterogeneity in the distribution of particles, which is clearly present in real systems, is not considered; (ii) a single particle size is considered in the model, while in reality the platinum particle size and also the inter-particle distances, both are subject to a natural distribution; (iii) the model assumes that the distribution of particles on the support is neither affected by favorable sites on the support, nor by any interactions between platinum particles; (iv) the model considers only the initial inter-particle distance before the degradation starts, even though the inter-particle distance (as for instance described earlier in this work for the Pt/Vulcan 3–4 nm material) is a function of time throughout the degradation process. Despite all these simplifications, the AID can be used as a rule of thumb to identify trends in the initially present inter-particle distance and thus for the likelihood for a Pt/C catalyst to suffer from coalescence and/or agglomeration under simulated start-stop conditions. This is for example demonstrated in Table 2, in which the calculated initial AID can be correlated with the extent of particle growth observed.

**Table 2:** Stability data of the three Pt/C catalysts of this study, as well as data from the four catalysts depicted in Figure 9 are summarized. Additionally, three further catalyst materials are included. Information about particle growth was deduced from IL-TEM, while the ECSA loss was determined via thin-film half-cell tests. The data from investigations of Schlögl et al. [49,67], Mayrhofer et al. [51], Hartl et al. [66] and Perez-Alonso et al. [68] were included in the comparison, and the AID was also calculated for these studies by using Equation 1. “rt” indicates experiments at room temperature.

material	publication (catalyst manufacturer)	particle diameter [nm]	Pt content [wt %]	BET support [m^2^·g^−1^]	aging protocol	ECSA loss [%]	calculated AID [nm]	particle growth

Pt/C 3 nm	Schlögl et al. [49,67] (TKK)	2.6	46	800	0.4–1.4 V_RHE_ 3600 cycles 1 V·s^−1^; rt 0.1 M HClO_4_	55	12	strong
Pt/C 5 nm (heat-treatment)	Mayrhofer et al. [51] (TKK)	4.8	51	800	0.4–1.4 V_RHE_ 3600 cycles 1 V·s^−1^; rt 0.1 M HClO_4_	31	28	none
	Schlögl et al. [49,67] (TKK)				1.3 V_RHE_ 16 h hold 348 K (75 °C) 0.1 M HClO_4_	24	<<28 (support shrinks)	strong
Pt/graph-C (graphitized)	Schlögl et al. [49,67] (TKK)	2-3	47	**—**	0.4–1.4 V_RHE_ 3600 cycles 1 V·s^−1^; rt 0.1 M HClO_4_	40	<<10	very strong
Pt/LSA-C I	Hartl et al. [66]	3	30	30	0.4–1.4 V_RHE_ 3600 cycles 1 V·s^−1^; rt 0.1 M HClO_4_	48	2	very strong
Pt/LSA-C II	Hartl et al. [66]	3	28	28	0.4–1.4 V_RHE_ 3600 cycles 1 V·s^−1^; rt 0.1 M HClO_4_	38	2	very strong
Pt/C 2.3 nm	Perez-Alonso et al. [68]	2.3	10	250	0.6–1.2 V_RHE_ 3000 cycles 0.2 V·s^−1^; rt 0.1 M HClO_4_	—	17	mild
Pt/C 2 nm	Schlögl et al. [49,67] (TKK)	2	20	**—**	0.4–1.4 V_RHE_ 3600 cycles 1 V·s^−1^; rt 0.1 M HClO_4_	58	—	strong
Pt/Vulcan 3–4 nm	current study (authors’ lab)	3–4	20	250	0.4–1.4 V_RHE_ 3600 cycles 1 V·s^−1^; rt 0.1 M HClO_4_	45	20	strong
Pt@HGS 1–2 nm (graphitized)	current study (authors’ lab)	1–2	20	1200	0.4–1.4 V_RHE_ 3600 cycles 1 V·s^−1^; rt 0.1 M HClO_4_	66	13	strong
Pt@HGS 3–4 nm (graphitized, heat-treatment)	current study (authors’ lab)	3–4	20	1200	0.4–1.4 V_RHE_ 5000 cycles 1 V·s^−1^; rt 0.1 M HClO_4_	19	48	none

**Figure 10:**
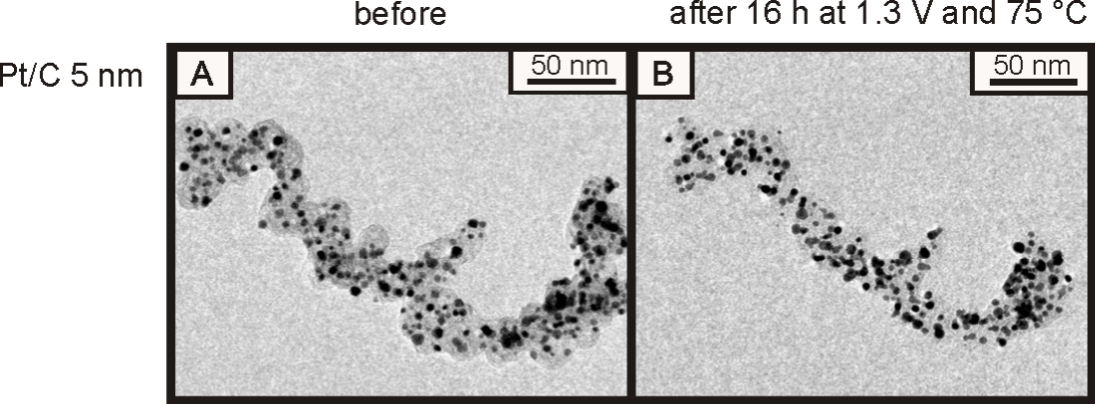
IL-TEM micrograph of Pt/C 5 nm subjected to 1.3 V_RHE_ at 348 K (75 °C) for 16 h in 0.1 M HClO_4_. A shrinkage of the carbon support due to carbon corrosion with successive decrease in inter-particle distances and coalescence can be observed. The images were reprinted with permission from [49]. Copyright 2011 Elsevier.

Almost all of the investigated materials included in Table 2 showed particle growth that was predominantly a result of coalescence, like in the case of the Pt/Vulcan 3–4 nm catalyst. Only for two materials, i.e., Pt/C 5 nm and Pt@HGS 3–4 nm, no observable particle growth after several thousands of potential cycles at room temperature was found. The calculated average inter-particle distances for these two particular catalysts are the largest among all depicted catalysts (i.e., about 28 nm for Pt/C 5 nm and about 48 nm for Pt@HGS 3–4 nm). Note that even though the AID of 28 nm is sufficient for the Pt/C 5 nm catalyst to prevent coalescence at room temperature, this is not the case at higher temperatures. Schlögl et al. [49] investigated the stability of this particular catalyst under constant potential (1.3 V_RHE_) and at 75 °C (Figure 10). At these elevated temperatures, carbon corrosion played a much more dominant role, and the shrinkage of the carbon support led to a decrease of the inter-particle distances and to successive coalescence as a secondary degradation process.

**Figure 11:**
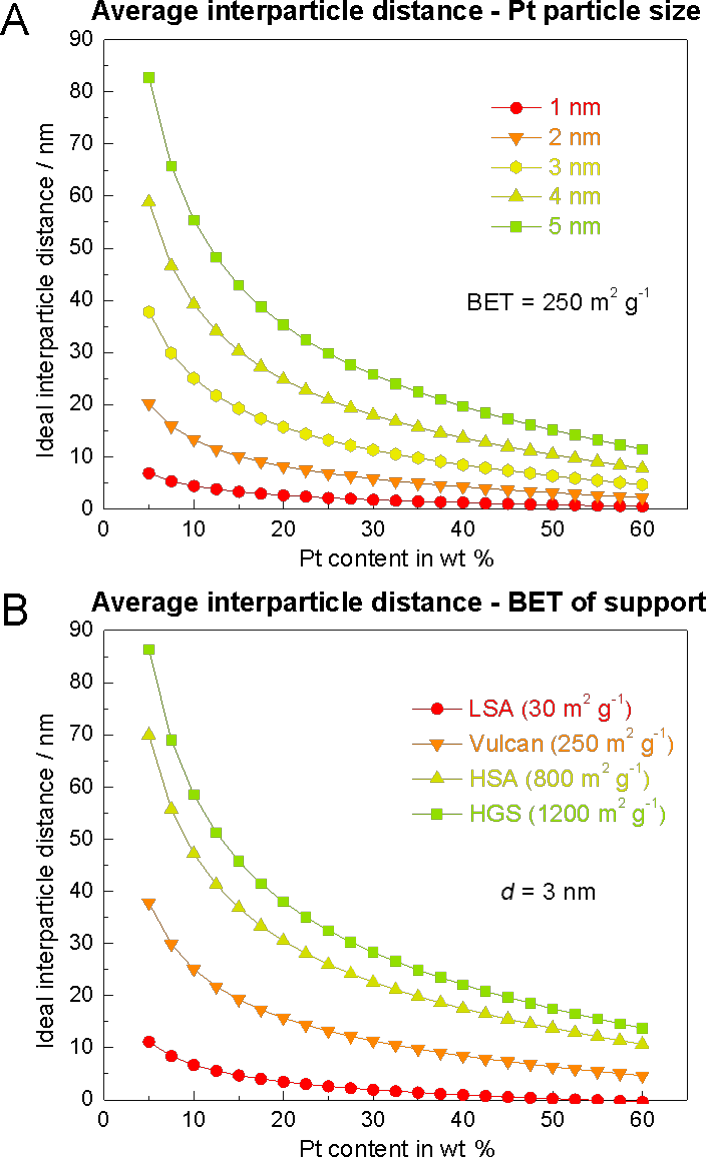
A) Dependence of the AID on platinum content for various platinum particle sizes, calculated for a Vulcan support (BET ca. 250 m^2^·g^−1^) using Equation 1. B) Dependence of the AID on platinum content for various specific support surface areas, calculated for a platinum particle diameter of 3 nm, by using Equation 1.

For all other catalysts depicted in Table 2 the inter-particle distances are much shorter and thus agglomeration and coalescence are already possible at room temperature without the need for strong carbon corrosion. Most remarkably, materials with very small inter-particle distances like Pt/graph-C (<<10 nm) or Pt/LSA-C I and II (2 nm both) (LSA-C I is a standard low surface area support, LSA-C II was described before) are characterized by the most severe particle growth. Even though the inter-particle distance is not the only important parameter to improve catalyst stability, an indication can be provided with the aid of Equation 1 on whether a catalyst with a known support, particle size and loading would offer sufficient inter-particle distances to make coalescence less likely to occur. Figure 11 illustrates how the AID depends on the platinum content for various platinum particle sizes (Figure 11A) and for various specific support surface areas (Figure 11B).

A value of zero for the inter-particle distance in the model would correspond to a densely packed monolayer of platinum nanoparticles on the carbon surface, while negative values indicate that this monolayer would even be exceeded and further particles would be stacked on top. It is noteworthy how strongly the AID decreases with decreasing platinum particle size in Figure 11. While a Pt/C catalyst (with BET of ca. 250 m^2^·g^−1^ as typical for standard Vulcan supports) with a particle size in the range of 1–2 nm is already in a regime of small inter-particle distances at low platinum loadings such as 10 wt %, catalysts with platinum particle sizes between 4–5 nm require loadings of 40 wt % or higher to reduce the average inter-particle distance to a similar degree. Moreover, the specific surface area of the carbon support has a strong impact on the AID, as shown in Figure 11B. It can be concluded from that figure that the low surface area supports (LSA) with a BET surface area of around 30 m^2^·g^−1^ (as explored by Hartl et al. [66]), despite providing an improved resistance against carbon corrosion, cannot sufficiently separate the platinum nanoparticles from each other. On the contrary, remarkably larger average inter-particle distances are offered by high surface area carbon supports (HSA), e.g., those for Pt/C 5 nm, 3 nm and 1–2 nm catalysts (all manufactured by TKK), and even more by HGS. This indicates that carbon supports with a high specific surface area have the advantage that higher platinum loadings (or smaller platinum particle sizes) can be used without ending up in inter-particle distances below the critical value at which coalescence is expected.

Apart from agglomeration and coalescence, it needs to be mentioned that small inter-particle distances may similarly enhance an Ostwald-ripening type of mechanism, because large concentrations of dissolved platinum can be expected in close proximity to other particles in regions with high particle density, acting as sites for redeposition. However, while coalescence due to initial contact occurs most likely during the initial stage of catalyst degradation, redeposition of dissolved platinum requires a significant dissolution first, so it probably has a stronger contribution to the overall surface area loss at a later degradation stage, as the findings of Hodnik et al. by using IL-SEM [64] may suggest.

Applying the above findings for the interpretation of the different degradation behavior of Pt/Vulcan 3–4 nm (AID = 20 nm), Pt@HGS 1–2 nm (AID = 13 nm) and Pt@HGS 3–4 nm (AID = 48 nm), helps to understand why the Pt@HGS 3–4 nm material is more stable towards agglomeration and coalescence. Even though Pt/Vulcan 3–4 nm has the same Pt content and a comparable particle size distribution, its inter-particle distance is comparatively small, which has its origin in the small specific surface area of the Vulcan carbon support compared to the HGS support. The Pt@HGS 1–2 nm catalyst on the other side, which again has the same Pt content as the Pt@HGS 3–4 nm catalyst along with the same carbon support, cannot reach a sufficient inter-particle separation due to the dispersion of the platinum into very small particles. The total number of platinum nanoparticles and thus their average distance is thus much smaller for the Pt@HGS 1–2 nm compared to the Pt@HGS 3–4 nm catalyst.

Overall under potential cycling conditions (between 0.4–1.4 V_RHE_ at 1 V·s^−1^) in 0.1 M HClO_4_ and at room temperature, three regimes for different AID, *l*, can be roughly identified: (i) *l* > 25 nm → no coalescence; (ii) 10 nm < *l* < 25 nm → some coalescence (extent dependent on quality and homogeneity of distribution of particles); and (iii) *l* < 10 nm → strong coalescence. Even though a distance of for instance 20 nm may appear as sufficiently large to prevent coalescence, it is crucial to understand that a certain fraction of nanoparticles will be in closer proximity, because the distance *l* is only an average value, which in reality corresponds to an inter-particle size distribution. In this case the fraction of smallest inter-particle distances (i.e., the number of particles in direct contact or close vicinity) is most likely the decisive parameter for the extent to which coalescence occurs. The above mentioned regimes should be seen as a first guideline, however they require confirmation and refinement on the basis of larger data sets. Note that as implied by the discussion of the degradation of Pt/C 5 nm at higher temperatures, the suggested regimes may change significantly under a different set of applied aging conditions.

### Effect of particle size

When a defined mass of platinum is dispersed on a given carbon support, decreasing the platinum particle size on a nanometer scale implicates (i) an alteration of electronic properties of platinum (ii) an increasing ECSA and (iii) decreasing inter-particle distances. These implications have a severe impact on the ORR activity as well as on the stability of Pt/C materials under fuel-cell operation conditions. The so called “particle/crystallite size effect” on the ORR (specific) activity was investigated in various studies [8–14] and thus is not at the focus of this work. However, it needs to be mentioned that the activity investigations on a broader basis of catalysts (Table 1 and Figure 2) in the current study confirm recent findings [12,14]. Changes in specific activity for carbon supported catalysts in a range of particle sizes between 1 and 5 nm were found to be within the error of the measurement, whereas a significant increase of specific activity is observed for Pt/C materials with particles larger than 5 nm. This observation was discussed and investigated in more detail in several recent works [12,14] and indicates that an increase in initial mass activity for supported catalysts is optimized by aiming for very small particle sizes.

**Figure 12:**
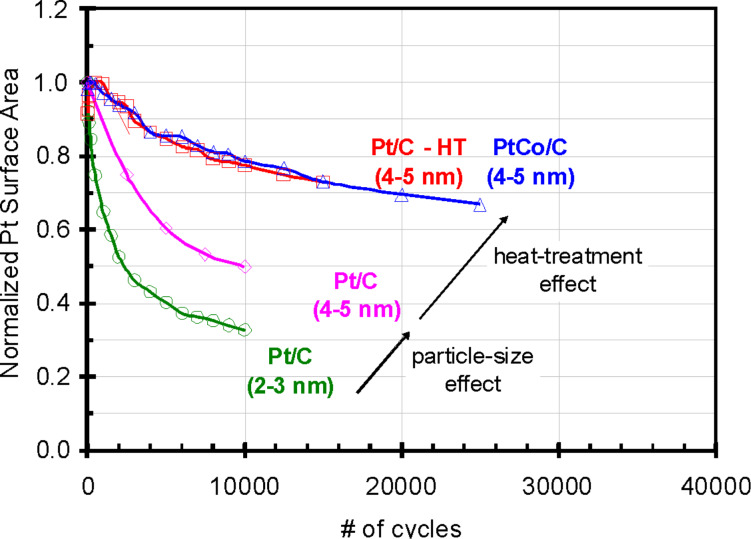
Impact of catalyst particle size and post-synthesis heat treatment on the normalized platinum surface area loss. The test was performed with H_2_ on the anode and N_2_ on the cathode at 80 °C, 100% RH. The cathode side of the membrane electrode assembly (MEA) is cycled between 0.6 and 1.0 V_RHE_ at 0.02 V·s^−1^. The graph was reproduced from [81] with permission. Copyright 2006 The Electrochemical Society.

However, the measurements on Pt@HGS 1–2 nm shown above or on the commercial Pt/C 1–2 nm catalyst (Figure S3 in Supporting Information File 1) demonstrate that catalysts with such small particles are not able to maintain their high initial ECSA, as they degrade very fast because of the rapid loss of the small particles. The low stability of small particles is reflected in Table 2, in which the most severe losses in ECSA are in general observed for catalysts with the smallest particle size. The comparison indicates that besides the AID, the particle size has an outstanding impact on catalyst stability, which is, for instance, also supported by the findings of Shao-Horn and coworkers [14] or Makharia et al. [81] (see below in Figure 12). According to these studies especially catalysts with a large fraction of platinum particles smaller than 2 nm suffer from the most severe surface area losses. A typical explanation for this is that with decreasing size of the platinum particles the curvature of the particles increases and thus the surface energy rises, which can impact the dissolution thermodynamics according to the so-called Gibbs–Thomson effect. This is expected to result in a negative shift of the Nernst potential of platinum dissolution for these particles compared to bulk platinum [15].

Recent observations on the dissolution of polycrystalline platinum with an electrochemical flow cell and online detection of dissolved platinum in the electrolyte via inductively coupled plasma mass spectrometry (ICP-MS) [82] have resulted in three major conclusions: (i) platinum dissolution is a transient process that occurs only when the potential changes cause a substantial change in the platinum surface state, while at constant potential conditions no platinum dissolution was observed; (ii) platinum dissolution can be separated into anodic and cathodic dissolution; and (iii) the amount of anodically dissolved Pt is linked to low-coordinated surface atoms and the amount of cathodically dissolved platinum is linked to the extent of oxidation. In the light of these observations the impact of particle size on platinum dissolution could be explained by the enhanced oxophilicity and thus increased oxide content at a given potential with decreasing particle size [11,14]. Additionally, the contribution of under-coordinated edge and corner sites to the overall surface area is rising for smaller particles. For platinum particles with a diameter of about 2 nm (assuming a cubo-octahedral shape) the surface already consists of more than 50% of under-coordinated edge and corner sites and their contribution drastically increases for even smaller particles, which is why especially Pt/C materials with a large fraction of particles below this size degrade so rapidly. Still, more quantitative investigations will be necessary to resolve the effect of particle size on dissolution, while particularly taking into account the increase in ECSA. Namely, a larger ECSA naturally implicates that more platinum atoms are available for both the desired oxygen reduction reaction as well as the undesirable dissolution of platinum.

The observation that catalysts with a significant amount of platinum particles smaller than 2 nm degrade much faster, suggests that the synthesis of stable Pt/C catalysts should rather aim for larger sizes. Considering that significantly larger particles lead to low mass activities, an average particle size of about 3–4 nm, as often employed anyway in commercial systems, seems to be indeed a reasonable choice for fuel cell materials. Moreover, a narrow particle size distribution, in order to neither waste platinum for unstable smaller particles nor for too large particles with low mass activity, are a precondition for the efficient utilization of platinum.

### Effect of thermal-treatment

Makharia et al. [81] performed an extensive study on the effect of thermal treatment, the larger average particle size (as a result of the heat treatment), and additionally on the intrinsic properties induced by alloying. They compared the surface area loss of four catalyst materials (Figure 12), namely Pt/C (2–3 nm), a “poorly dispersed” Pt/C (4–5 nm), a “well-dispersed” heat-treated Pt/C-HT (4–5 nm) catalyst and a platinum-alloy catalyst, i.e., PtCo/C (4–5 nm). All catalysts were subjected to the same single-cell aging test, namely potential cycling between 0.6 and 1.0 V (at 80 °C, 100% RH, 0.02 V·s^−1^).

First, the findings of Makharia et al. [81] indicate that the improvement of the stability of the alloy catalyst is due to a “heat-treatment effect” (direct or indirect) and not due to an improved intrinsic material stability of the alloy, as both the Pt/C-HT (4–5 nm) and the PtCo/C (4–5 nm) material show an almost identical surface area loss. Furthermore, it is remarkable that their thermally treated Pt/C-HT (4–5 nm) catalyst is significantly more stable than the Pt/C (4–5 nm) reference material, which was synthesized without a thermal-treatment step. Therefore, the stabilization due to thermal treatment cannot merely be attributed to an increase in average particle size, but also other aspects contribute to the effect of the thermal treatment. However, an explanation for the origin of the improved stability upon thermal treatment was not provided. In this context, Shao-Horn et al. [15] emphasized that it is important to take into account the complete particle size distribution and not only the average particle size when comparing thermally treated and non-treated materials. In particular the fraction of smaller particles of Pt/C catalysts is decreased significantly during thermal treatment, as small platinum particles will presumably migrate on the carbon support and coalesce into larger particles. Thus the fraction of platinum particles smaller than 2 nm in the overall particle size distribution will be smaller than for other Pt/C catalysts of comparable average particle size. Since those smaller particles are expected to dissolve faster under degradation conditions, the improved stability of the thermally treated catalyst materials with a smaller fraction of small particles could be explained with the particle size effect, as described above, however, while taking the distribution into account.

This is well in line with our investigations of the Pt/Vulcan 3–4 nm, Pt@HGS 1–2 nm and Pt@HGS 3–4 nm catalysts. However a further effect that contributes to the thermal-treatment effect, which is linked again to the inter-particle distance, was not considered so far. The Pt@HGS 3–4 nm catalyst is produced from the Pt@HGS 1–2 nm material in the process of a thermal-treatment step up to 900 °C. As can be seen from the comparison of the two Pt@HGS materials, not only the particle size increases from 1–2 nm to 3–4 nm during the thermal treatment, but also the AID changes from about 13 nm to about 48 nm after thermal treatment (see magnified insets for both materials in Figure 5). This is probably because particles in close proximity to each other (or even in direct contact) will preferentially already coalesce during the thermal treatment step. Therefore, the fraction of small inter-particle distances will decrease substantially and thus degradation due to agglomeration in general and coalescence due to initial contact in particular will be less likely to occur under fuel-cell operation conditions. The comparison of the degradation of the Pt/Vulcan 3–4 nm catalyst and the Pt@HGS 3–4 nm material as depicted in Figure 5, Figure 6 and Figure 7 supports this interpretation of the thermal-treatment effect due to different inter-particle distances. The Pt/Vulcan 3–4 nm catalyst does not only have a similar average particle size as the Pt@HGS 3–4 nm catalyst, but also a comparable particle size distribution, thus the number of particles smaller than 2 nm is about the same for both materials. However, the particle separation is improved for the thermally treated Pt@HGS 3–4 nm, and this catalyst is also much more stable toward coalescence compared to the Pt/Vulcan 3–4 nm catalyst. Similarly, the commercial Pt/C 5 nm catalyst with an AID of 28 nm (Figure 9 and Table 2) results from a thermal treatment of the Pt/C 3 nm material with an AID of ca. 12 nm [67]. Again, the non-treated catalyst shows significant coalescence among other degradation mechanisms, contrary to the thermally treated catalyst that shows no signs of coalescence during potential cycling at room temperature. It is noteworthy that the effect of thermal treatment was recently also addressed by Stephens et al. [83], who suggested that the heating results in a smoothening of the catalyst surface and the removal of defects or under-coordinated sites, which are most prone to corrosion. It can therefore be summarized that a decrease in the fraction of smallest particles in the particle size distribution, as well as a decrease of the smallest inter-particle distances, eventually together with a decrease of the number of under-coordinated sites, all contribute to the increased stability of thermally treated Pt/C catalysts. It is remarkable that the Pt/C 5 nm catalyst and the Pt@HGS 3–4 nm catalyst, i.e., the only two materials that were synthesized through a thermal treatment, are those with the lowest relative ECSA loss. This emphasizes that the thermal-treatment step to high temperatures is indeed a valuable option to improve the stability of the material, as long as the particle size can be kept in the desirable range for a high mass activity.

### Effects of carbon support: graphitization, specific surface area, surface structure, and pore confinement

While the activity for the ORR (Table 1 and Figure 2) is not significantly influenced by the type of carbon support for the tested Pt/C catalysts in thin-film RDE experiments (a study of mass transport effects would require fuel cell measurements), strong differences in the stability of the catalysts become obvious (see Table 2). Especially the small losses in ECSA for the catalysts with graphitized support (Pt/graph-C) and low surface area supports (Pt/LSA-C I and Pt/LSA-C II) all depict quite good stabilities considering their comparatively small platinum particle sizes, and the initial agglomeration and coalescence due to the very small inter-particle distances as a consequence of their low specific support surface areas. This observation is in good agreement with literature reports that emphasize the positive effect of graphitization on the resistance against carbon corrosion [23,67,84]. The effect of the surface structure of the carbon supports for fuel cell catalysts was demonstrated by Reetz et al. [85] in a separate study. Platinum particles have a lower tendency to agglomerate during the synthesis process of a Pt/C catalyst, if the support surface depicts a pronounced tortuosity. In general micro- and mesopores in carbon support materials are believed to act as a kind of physical barrier [86]. The concept of using the support surface structure to separate the platinum particles can be extended to the extreme case of a 3D interconnected mesoporous network. Here the platinum particles are confined to the mesoporous structure that provides various hosting sites. These hosting sites also play a crucial role during the heat-treatment step and the control of the particle growth via confinement of Pt in pores [71]. Overall, the Pt@HGS 3–4 nm material can thus be considered as an extension of the graphitized catalysts offering not only a high degree of graphitization, but also a high specific surface area and stabilization by a confinement to the pores, which results in a highly active and stable catalyst.

The combination of the graphitization, the thermal-treatment effect and the pore-confinement opens up also an interesting option for highly stable and active Pt-alloy fuel cell catalysts. The disadvantage of a pronounced thermal-treatment step for standard supports, i.e., an increased particle size and thus decreased ECSA and mass activity, generally limits the beneficial effects of alloying on the specific activity. The extraordinary control of the particle growth in the HGS support can help to circumvent this issue and thus offers an excellent opportunity for the synthesis of a new class of high-temperature annealed platinum-alloy catalysts with high ECSA and stability.

## Conclusion

Detailed investigations on the activity and the stability of electrochemical half-cells with three selected Pt/C catalysts and comparisons with a library of further Pt/C materials provided insights into several parameters that influence the performance of the catalysts. In particular identical location electron microscopy revealed how the material design can impact the degradation behavior of electrocatalysts under accelerated-aging conditions. Coalescence of not sufficiently well separated platinum nanoparticles on the support often plays a major role, especially at the initial stage of catalyst degradation. The average inter-particle distance (AID), which depends on the surface area of the carbon support, the platinum content of the catalyst, the platinum particle size as well as the quality of the distribution of the platinum particles on the support, was derived as a simple quantity that aids to evaluate an eventual impact of coalescence. Moreover, it was demonstrated that the platinum particle size is one of the most dominant parameters for the stability of the Pt/C catalyst. While a surprisingly small influence of the platinum particle size on the specific activity of the catalyst was found for Pt/C materials based on supported nanoparticles in a range of 1–5 nm, a strong impact on the stability is evident especially when large fractions of the platinum particles are smaller than 2 nm. A thermal treatment of Pt/C catalysts is quite beneficial in this aspect, as it reduces the amount of the smallest particles, which would be otherwise lost during operation, and it also leads to larger AIDs and thus a better separation of the resulting larger particles. In order to optimize carbon-supported catalysts for both, activity and mass activity, it is thus necessary to thoroughly consider all of these aspects. In particular the transformation of the catalyst material during long-term operation should already be taken into account at the stage of materials design.

## Experimental

The details of the synthesis and characterization of the HGS support as well as the pore-confinement of the Pt nanoparticles can be found in our previous publication [71]. The electrochemical procedures are shortly summarized in the following, as they have been previously described more extensively [12,16,40,72–73]. All electrochemical measurements were performed in a three-compartment electrochemical Teflon cell, while using a rotating disk electrode (RDE) setup, a radiometer analytical rotation controller and a Gamry Reference 600 potentiostat. A graphite rod was used as counter electrode and an Ag/AgCl electrode (Metrohm) as reference. However, all potentials were referenced to the reversible hydrogen electrode (RHE), which was determined prior to every measurement. The rotator, the potentiostat and the gas flow were automatically regulated by using a LabVIEW based software that was developed in-house [87]. The Ag/AgCl electrode compartment was separated from the main compartment of the cell via a Nafion membrane to prevent the tested materials from contamination with chlorides during the activity and stability tests [88]. The working electrode was made of a Teflon tip with an embedded glassy carbon disc (5 mm diameter, 0.196 cm^2^ geometrical surface area) onto which the catalyst suspension was directly pipetted to form films for the activity or macroscopic stability tests, or on which a gold finder grid was contacted for identical location electron microscopy measurements. In the latter case the catalyst was pipetted on the grid after contacting. The standard electrolyte volume was about 150 mL and all tests were performed at room temperature. The electrolytes, i.e., 0.1 M HClO_4_ and 0.1 M H_2_SO_4_, were prepared with ultrapure water (18 MΩ·cm, ELGA) and conc. HClO_4_ (Merck, Suprapur) or conc. H_2_SO_4_ (Prolabo, Normapur). The resistance of the solution was taken into account by positive feedback compensation so that the residual uncompensated resistance was less than 4 Ω in all measurements. Catalyst suspensions were made by dispersing the catalyst powders ultrasonically in ultrapure water for at least 30 minutes.

### Activity measurements

Activity measurements were carried out for different loadings of catalyst on the working electrode to exclude mass transport limitations as a result of a too thick catalyst film. Freshly sonicated catalyst suspensions (at least 5 minutes prior to application) were pipetted onto the glassy carbon disc and dried in air or under mild vacuum. Typical loadings for the commercial and self-made Pt/C materials were in the range of 2.5 to 40 µg_Pt_·cm^−2^ at the electrode in order to obtain thin and well-dispersed catalyst films. Typical loadings for Pt-black to achieve good films were in the range of 40–80 µg_Pt_·cm^−2^. The materials were subjected to potential cycles before activity and ECSA measurements in order to clean the catalyst surface until stable cyclic voltammograms were obtained. Significantly more cleaning cycles were necessary for the Pt@HGS 3–4 nm catalyst (typically 200–300 cleaning cycles, 0.05–1.35 V_RHE_, 0.2 V·s^−1^) as a consequence of the heat-treatment procedure during the synthesis. General guidelines for activity measurements were followed as described previously [12,72–73]. Specific activities were calculated from the positively directed scan of the RDE polarization curves at 0.9 V_RHE_, a rotation rate of 1600 rpm and a scan rate of 0.05 V·s^−1^.The RDE polarization curves were corrected for capacitive processes to consider only the current that was related to the ORR. For this purpose, a cyclic voltammogram recorded with the same scan rate and potential window but in argon-saturated electrolyte was subtracted from the ORR polarization curves.

The platinum surface area was determined by electrochemical oxidation of pre-adsorbed carbon monoxide (CO-stripping). In each CO-stripping measurement, carbon monoxide was adsorbed on platinum in a potential region (e.g., 0.05 V_RHE_) at which carbon monoxide is stable at the surface, until the saturation coverage was reached. Afterwards, the electrolyte was purged with argon until all carbon monoxide was removed from the electrolyte, while still the same potential was held. Finally, the adsorbed carbon monoxide was oxidized electrochemically in stagnant electrolyte, and the charge corresponding to the oxidation was measured by the area of the oxidation peak. Mass activities were calculated be multiplying the specific activity and ECSA, which was determined independently with several CO-stripping experiments for at least three different catalyst loadings at the working electrode.

### “Macroscopic stability tests”

The macroscopic stability tests, i.e., thin-film degradation tests, for the three Pt/C materials were performed on thin films with the same catalyst loading (i.e., the same amount of catalyst on the working electrode) for each of the three materials, namely 30 µg_Pt_·cm^−2^. The tests were done in 0.1 M HClO_4_, at room temperature and without rotation. The accelerated-aging protocol consisted of 10800 potential cycles (triangular wave) between 0.4 and 1.4 V_RHE_ with a sweep rate of 1 V·s^−1^. The surface of the catalysts was not subjected to potential cycles for cleaning before the test, to report the surface area changes from the start. CO-stripping was used to monitor changes in ECSA after 0, 360, 1080, 2160, 3600, 5400, 7200 and 10800 potential cycles.

### “Nanoscale stability test”

The catalyst suspensions as used for the thin-film degradation experiments were diluted by a factor of 5 with ultrapure water. A drop of the suspension was loaded on the front side of a gold finder grid (NHA7, Plano) coated with a holey carbon film (Quantifoil R2/2). To avoid high catalyst loadings, which can result in overlapping catalyst particles, the drop was absorbed off the grid with a tissue after a few seconds. IL-SEM and IL- STEM experiments were performed with a Hitachi S-5500 ultra-high resolution cold field emission scanning electron microscope at 30 kV, which allows SEM as well as STEM measurements. All other IL-TEM measurements were carried out with a JEM-2200FS (Jeol, Japan) transmission electron microscope, operated at an acceleration voltage of 200 kV. The catalyst deposited on the TEM grid was treated electrochemically by immobilizing the gold finder grid on the glassy carbon disc working electrode with the help of a Teflon cap as reported previously [16,40]. The aging procedures were designed analogous to the macroscopic degradation study. Tests after 0 and 3600 or 5000 degradation cycles between 0.4 and 1.4 V_RHE_ with a sweep rate of 1 V·s^−1^ without rotation were applied in 0.1 M HClO_4_ saturated with argon. The measurements were again performed at room temperature and IR-compensation was achieved via positive feedback. CO-stripping experiments were not carried out between the degradation cycles for the identical location studies, because it is not possible to determine the area of such low amounts of catalyst as dispersed on the TEM finder grid. All particle size distributions were determined from the 2D IL-TEM and IL-STEM images. As the particles are not spherical, the shape was approximated with ellipses. A diameter corresponding to an ideal circle was calculated for every single particle from the area obtained from the ellipse, and this was used to calculate the average spherical diameter. Further information regarding the basic identical location electron microscopy method can be found in the literature [40,51,71].

## Supporting Information

Supporting Information features a schematic illustration of the most important steps in the synthesis process of HGS, Pt@HGS 1–2 nm and Pt@HGS 3–4 nm. TEM images of reference materials, activity data in sulphuric acid, thin-film degradation tests on a commercial Pt/C 1–2 nm catalyst as well as further IL-TEM data are also available together with the derivation of the equation for the average inter-particle distance.

File 1 Further experimental data.
