# Supplementary material for: Design criteria for stable Pt/C fuel cell catalysts
Source: Beilstein J Nanotechnol. 2014 Jan 16;5:44–67. doi: 10.3762/bjnano.5.5 (PMC3943646; doi:10.3762/bjnano.5.5)
Supplement: File 1 — Further experimental data. [file Beilstein_J_Nanotechnol-05-44-s001.pdf]

Supporting Information

for

Design criteria for stable Pt/C fuel cell catalysts

Josef C. Meier*[1,‡], Carolina Galeano[2,‡], Ioannis Katsounaros[1], Jonathon Witte[1], Hans J. Bongard[2], Angel A. Topalov[1], Claudio Baldizzone[1], Stefano Mezzavilla[2], Ferdi Schüth[2] and Karl J. J. Mayrhofer*[1,§]

Address: [1]Department of Interface Chemistry and Surface Engineering, Max-Planck-Institut für Eisenforschung GmbH, Max-Planck-Strasse 1, 40237 Düsseldorf, Germany; [2]Department of Heterogeneous Catalysis, Max-Planck-Institut für Kohlenforschung, Kaiser-Wilhelm-Platz 1, 45470 Mülheim an der Ruhr, Germany

E-mail: Josef C. Meier* - meier@mpie.de; Karl J. J. Mayrhofer* -mayrhofer@mpie.de

[§]Tel.: +49 211 6792 160, Fax: +49 211 6792 218

[‡]These authors contributed equally.

Further experimental data

Synthesis of HGS, Pt@HGS 1–2 nm and Pt@HGS 3–4 nm:

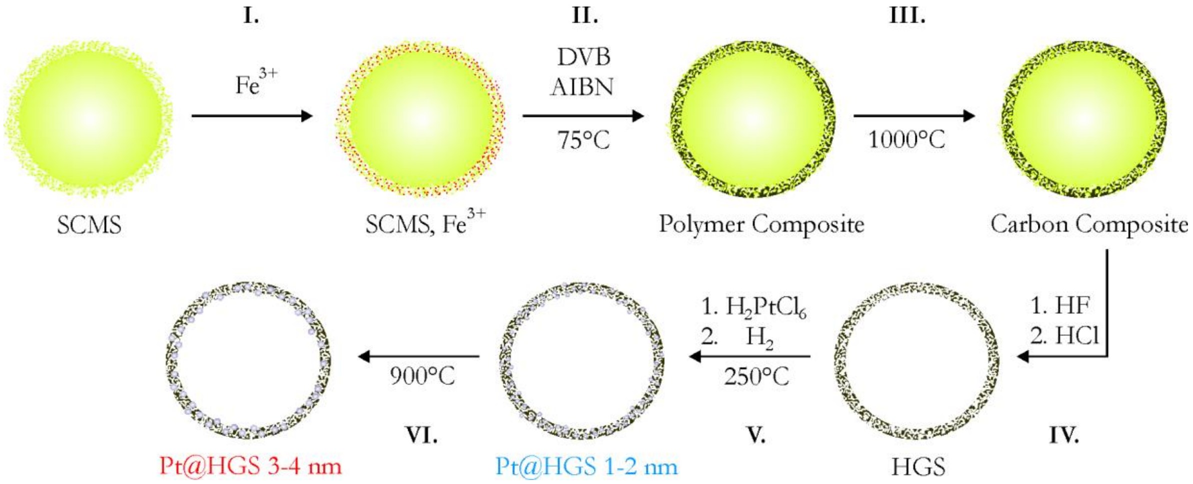

Scheme S1: Synthesis procedure of HGS, Pt@HGS 1–2 nm and Pt@HGS 3–4 nm.

The synthesis of HGS and Pt@HGS has been described in our earlier work [1]. However the most important steps in the synthesis procedure are summarized in Scheme S1. The synthesis of the hollow graphitic sphere carbon support is carried out by nanocasting starting from an exotemplate with a solid silica core and a mesoporous silica shell (SCMS) [2]. In a first step (I.) the exotemplate is impregnated with an iron(III) salt, $Fe(NO_3)_3{\cdot}9H_2O$, which is latter needed as a catalyst precursor for graphitization. Other transition metal salts such as nickel or cobalt salts can also facilitate graphitization, however, they proved to be less effective. Applying the incipient wetness method the exotemplate is incorporated with a carbon source, divinylbenzene (DVB), and a radical starter, azo-bis-isobutyronitril (AIBN), for the polymerization process. The formation of the polymer, poly(divinylbenzene) (PDVB), occurs under modest heating, thus forming a

composite material together with the silica exotemplate (II.). The subsequent carbonization of the polymer in an inert atmosphere at 1000 °C results in a carbon composite material (III.). It is during this step that graphitic domains are formed assisted by iron acting as graphitization catalyst. Finally dissolving of the silica template with HF 10 vol % in water (alternatively also with NaOH) and a removal of iron with concentrated HCl followed by some cleaning steps in ultrapure water and ethanol provides the hollow graphitic sphere support (IV.).

The incorporation of platinum into the mesoporous network of the spheres is achieved via ultrasound assisted incipient wetness impregnation of a solution of hexachloroplatinic acid, $H_2PtCl_6 \cdot xH_2O$, and successive reduction with H_2 at 250 °C (V.). This method has the advantage, that after reduction there is only a small population of particles at the external surface of the support, while the majority of particles is incorporated into the mesoporous shell. The catalyst material, which was synthesized also for electrochemical characterization, has a platinum loading of 20 wt % and platinum particles predominantly in the range of 1–2 nm. It is hereafter denoted as Pt@HGS 1–2 nm.

If the Pt@HGS 1–2 nm material is furthermore subjected to a thermal treatment under argon atmosphere (annealing rate: 5 $°C \cdot min^{-1}$) up to ca. 900 °C (for several hours) a catalyst with an average platinum particle size of 3–4 nm is obtained (VI.). This thermally treated material is hereafter abbreviated as Pt@HGS 3–4 nm and is characterized by a platinum content of 20 wt %.

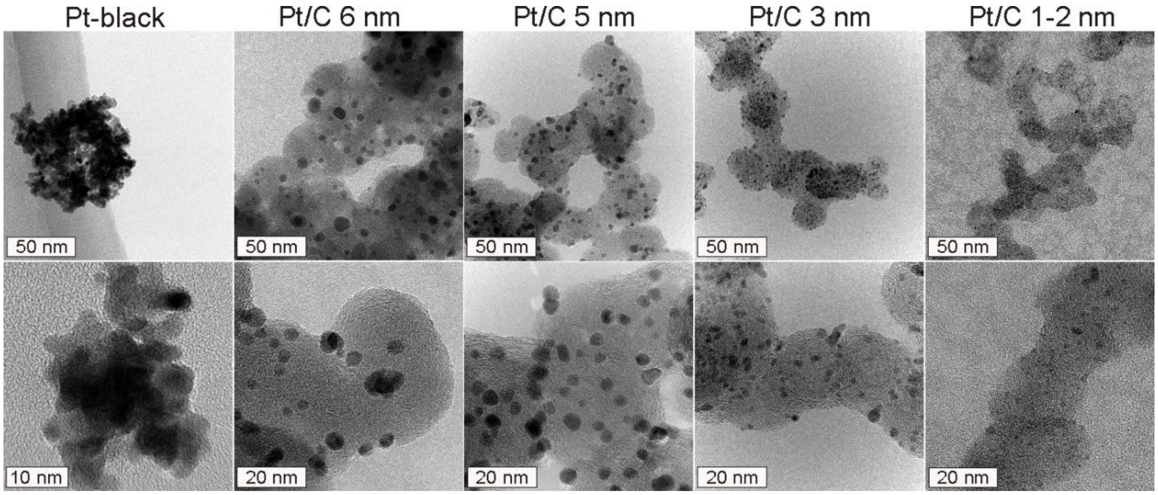

Figure S1: TEM images of reference catalysts (besides of Pt-poly) used for activity comparisons.

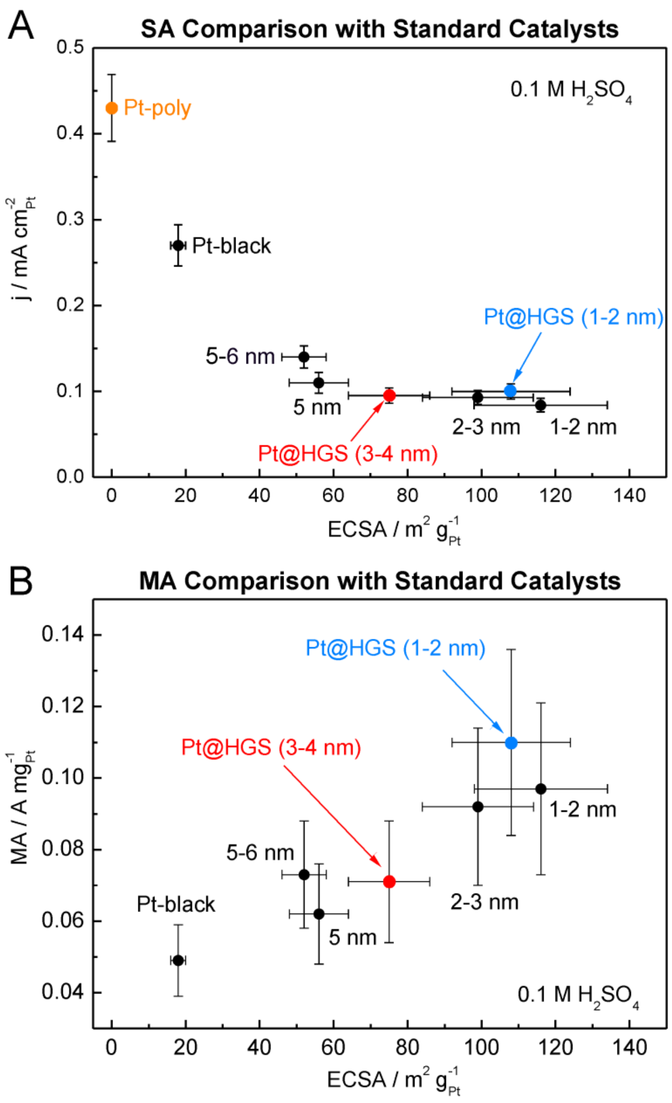

Figure S2: A) The plot of specific activity versus electrochemical active surface area of platinum, illustrates the particle size dependent changes in activity in 0.1 M H_2SO_4. Activities were determined in the anodic scan at 0.9 V_{RHE} at room temperature with a scan rate of 0.05 $V \cdot s^{-1}$ and 1600 rpm. B) Mass activity versus electrochemical active surface area. Background correction for capacitive currents and IR-compensation via positive feedback was performed accordingly for all measurements. SA, MA and ECSA values are based on measurements at various loadings on the working electrode for each catalyst in order to exclude mass transport limitations. The same general trends are observed as for 0.1 M $HClO_4$ (see Figure 1 in the main article). The absolute activity values are lower, which is attributed to the well known effect of adsorption of sulfates/bisulfates to the platinum surface.

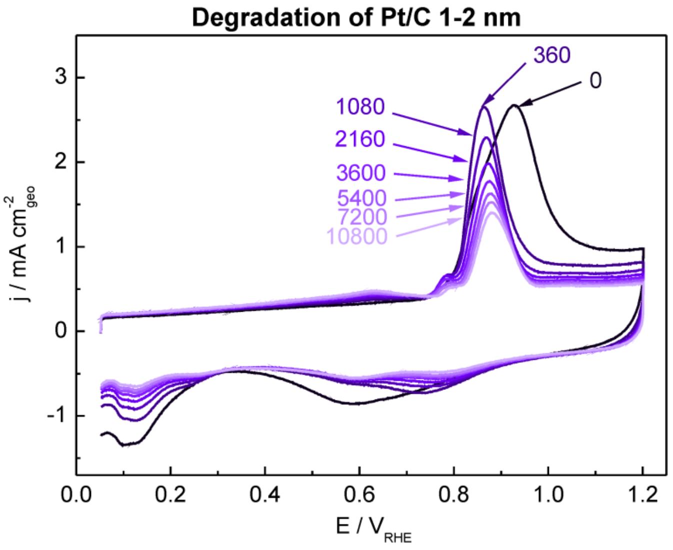

Figure S3: Electrochemical carbon monoxide monolayer oxidation curves (CO-stripping curves) after 0, 360, 1080, 2160, 3600, 5400, 7200 and 10800 degradation cycles for Pt/C 1–2 nm (10 wt % platinum, TKK, HSA support with BET of ca. 800 $m^2 \cdot g^{-1}$). The degradation cycles (not shown) are performed between 0.4 and 1.4 V_{RHE} with a scan rate of 1 $V \cdot s^{-1}$ at room temperature in argon-saturated 0.1 M $HClO_4$ (without rotation). CO-stripping voltammograms are recorded between 0.05 and 1.2 V_{RHE} at a scan rate of 0.05 $V \cdot s^{-1}$. The characteristic shape changes as described for the Pt@HGS 1–2 nm catalyst in the main article are also reflected for this material.

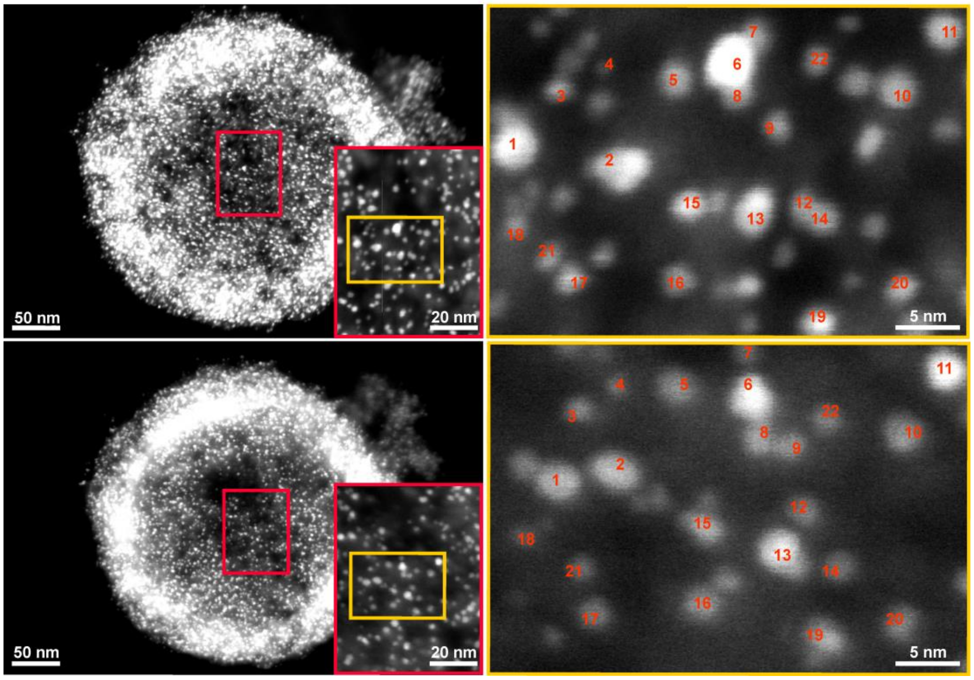

Figure S4: Identical location dark field scanning transmission electron microscopy (IL-STEM) of Pt@HGS 3–4 nm after 0 (top) and after 3600 (bottom) potential cycles between 0.4 and 1.4 V_{RHE} in 0.1 M $HClO_4$ at a scan rate of 1 $V \cdot s^{-1}$ at room temperature. The red rectangular shapes highlight subregions in the micrograph, which are enlarged for a better visualization of the platinum particles. The yellow rectangular shapes are a further magnification. They often enable direct identification of particles again after 3600 cycles, despite of the complexity due to the structure of the support. The numbers indicate the particles, which are probably the same. It becomes clear that some particles slightly change position.

AID-equation:

$$l = \sqrt{\frac{\pi}{3\sqrt{3}} \cdot 10^{-3} \cdot \rho_{Pt} \cdot \left(\frac{100 - L_{\mathrm{Pt}}}{L_{\mathrm{Pt}}}\right) \cdot A_{\mathrm{S}} \cdot d^{3}} - d$$

With l being the "average inter-particle distance" or short AID (nm), ρ_{Pt} the density of platinum (21.45 g·cm^{-3}), L_{Pt} is the platinum content (wt %), A_{s} is the specific surface area of the support (m^{2}·g^{-1}), and d is the platinum particle diameter (nm).

The average inter-particle distance equation is derived as described in the following on the basis of simple geometric considerations. For the calculation all platinum particles are assumed to have the same platinum particle diameter d and the same inter-particle distance l, which is referring to the distance of the surfaces of the platinum particles (Figure S5).

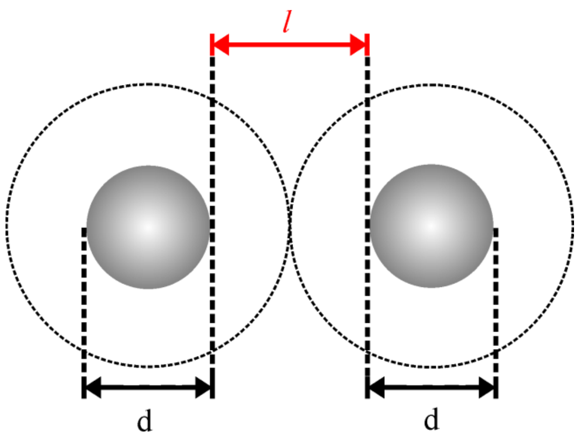

Figure S5: Two neighboring platinum particles with particle diameter *d* and inter-particle distance *l*.

The carbon support surface area A_{Support} is approximated by assuming a 2D surface area onto which the platinum particles are perfectly distributed, i.e., with equal distance to each other (Figure S6).

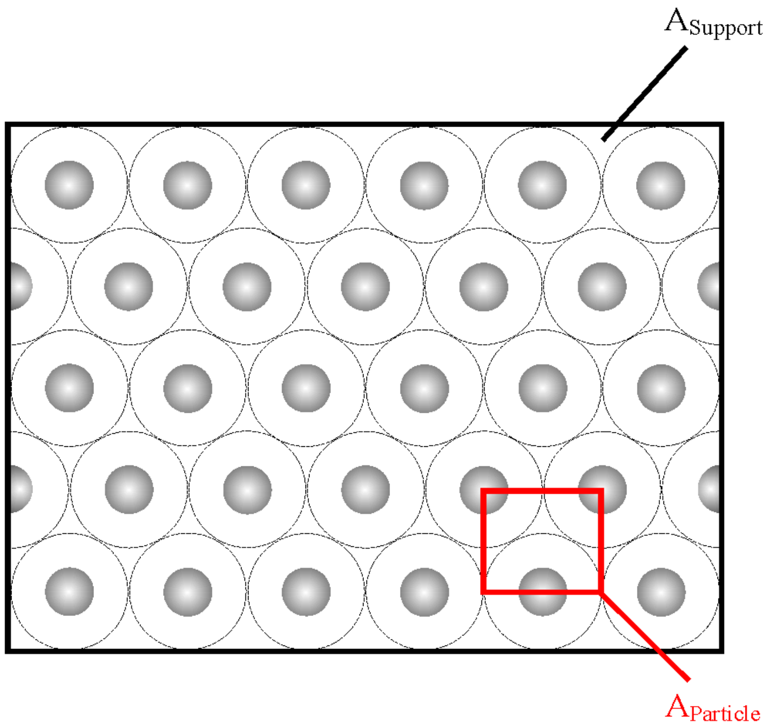

Figure S6: The platinum particles are assumed to be equidistant to each other on the carbon support that which is assumed to be two-dimensional. The surface of the carbon support, A_{Support}, is the area in the black rectangle, while the area of the red rectangle is the area, A_{Particle}, available for a single particle.

The area enclosed by the black rectangle corresponds to the area of the carbon support A_{Support} while the red rectangle reflects the fraction of the carbon support surface area for a single platinum particle and is denoted in the following with A_{Particle}. A_{Particle} can be expressed as a function of the particle diameter d and the inter-particle distance l. The geometric correlations can be deduced from Figure S7, which visualizes the dependence of the width x and the height y of the red rectangle on the particle diameter and the inter-particle distance.

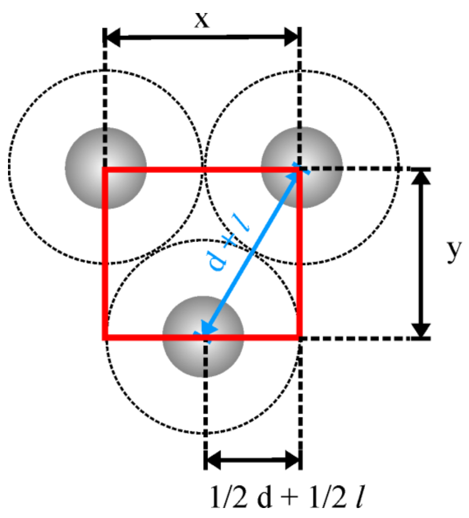

Figure S7: The fraction of the carbon support surface area that belongs to a single platinum particle A_{Particle} can be expressed as a function of particle diameter *d* and inter-particle *l* distance by using simple geometric relationships.

The area that belongs to a single platinum particle is thus given by:

$$A_{\text{Particle}} = x \cdot y$$

By using the geometric information $x = d + l$ and $y = \frac{\sqrt{3}}{2} \cdot (d + l)$ one obtains:

$$A_{\text{Particle}} = \frac{\sqrt{3}}{2}(d + l)^2$$

The area of the carbon support A_{Support} equals the area per platinum particle A_{Particle} multiplied by the total number of platinum particles *Z*:

$$A_{\text{Support}} = Z \cdot A_{\text{Particle}}$$

The total number of particles is given by the total mass of platinum divided by the mass of platinum of a single particle:

$$Z = \frac{m_{\text{Pt}}}{m_{\text{Particle}}}$$

The mass of a single platinum particle can also be expressed by the product of density of platinum ρ_{Pt} and the volume of the single particle V_{Particle}:

$$m_{\mathrm{Particle}} = \rho_{\mathrm{Pt}} \cdot V_{\mathrm{Particle}} \,.$$

Assuming spherical geometry the mass of a single platinum particle thus corresponds to:

$$m_{\mathrm{Particle}} = \rho_{\mathrm{Pt}} \cdot \frac{1}{6} \cdot \pi \cdot d^3$$

The total mass of platinum in the catalyst corresponds to the content of platinum L_{Pt} in wt % times the mass of the Pt/C catalyst, m_{Catalyst}:

$$m_{\mathrm{Pt}} = L_{\mathrm{Pt}} \cdot m_{\mathrm{Catalyst}}$$

Combining the equations for the total mass of platinum and the mass of a single platinum particle the total number of platinum particles Z is:

$$Z = \frac{L_{\mathrm{Pt}} \cdot m_{\mathrm{Catalyst}}}{\frac{1}{6} \cdot \rho_{\mathrm{Pt}} \cdot \pi \cdot d^3}$$

Inserting the term for Z in the correlation from above, $A_{\mathrm{Support}} = Z \cdot A_{\mathrm{Particle}}$ leads to:

$$A_{\mathrm{Support}} = \frac{6 \cdot L_{\mathrm{Pt}} \cdot m_{\mathrm{Catalyst}}}{\rho_{\mathrm{Pt}} \cdot \pi \cdot d^3} \cdot A_{\mathrm{Particle}}$$

By using the term from above for the area per single platinum particle gives:

$$A_{\text{Support}} = \frac{6 \cdot L_{\text{Pt}} \cdot m_{\text{Catalyst}}}{\rho_{\text{Pt}} \cdot \pi \cdot d^3} \cdot \frac{\sqrt{3}}{2} \cdot (d + l)^2$$

Solving the equation provides the average inter-particle distance *l*:

$$l = \sqrt{\frac{\pi}{3\sqrt{3}} \cdot \rho_{\text{Pt}} \cdot \left(\frac{1}{L_{Pt}}\right) \cdot \frac{A_{\text{Support}}}{m_{\text{Catalyst}}} \cdot d^3} - d$$

The support surface area A_{Support} is given by the product of specific carbon support surface area and the mass of the carbon support m_{Support}:

$$A_{\text{Support}} = A_{\text{S}} \cdot m_{\text{Support}}$$

For a pure Pt/C fuel cell catalyst the mass of the support is linked to the mass of the Pt/C catalyst via:

$$m_{\text{Support}} = m_{\text{Catalyst}} \cdot (100 - L_{\text{Pt}})$$

By using these relationships one obtains the inter-particle distance *l* as an intensive variable:

$$l = \sqrt{\frac{\pi}{3\sqrt{3}} \cdot \rho_{\mathrm{Pt}} \cdot \left(\frac{100 - L_{\mathrm{Pt}}}{L_{\mathrm{Pt}}}\right) \cdot A_{\mathrm{S}} \cdot d^3} - d$$

For a more comfortable use concerning common units to obtain the average inter-particle distance in nm (entering ρ_{Pt} = 21.45 g·cm^{-3}, the platinum loading L_{Pt} in percentage, the specific carbon surface area in m^2·g^{-1} and the particle diameter in nm) the equation transforms to:

$$l = \sqrt{\frac{\pi}{3\sqrt{3}} \cdot 10^{-3} \cdot \rho_{\mathrm{Pt}} \cdot \left(\frac{100 - L_{\mathrm{Pt}}}{L_{\mathrm{Pt}}}\right) \cdot A_{\mathrm{S}} \cdot d^3} - d$$

The equation directly reflects the dependence of the average inter-particle distance on the three parameters specific carbon support surface area, platinum loading, and the parameter with the strongest impact, the platinum particle diameter.

References

[1] Galeano, C.; Meier, J. C.; Peinecke, V.; Bongard, H.; Katsounaros, I.; Topalov, A. A.; Lu, A.; Mayrhofer, K. J. J.; Schüth, F. *J. Am. Chem. Soc.* **2012,** *134,* 20457–20465.

[2] Buchel, G.; Unger, K. K.; Matsumoto, A.; Tsutsumi, K. *Adv. Mater.* **1998,** *10,* 1036.
